# A lava-inspired proteolytic enzyme therapy on cancer with a PEG-based hydrogel enhances tumor distribution and penetration of liposomes

**DOI:** 10.1186/s12951-024-02468-7

**Published:** 2024-05-02

**Authors:** Jiaojiao Li, Dandan Mi, Rujing Wang, Yuke Li, Mengnan Zhao, Sanjun Shi

**Affiliations:** State Key Laboratory of Southwestern Chinese Medicine Resources, School of Pharmacy, Chengdu University of Traditional Chinese Medicine, Chengdu, 611137 China

**Keywords:** Proteolytic enzyme therapy, Tumor distribution of nanomedicine, Enzyme-assisted crosslinking, Hydrogel

## Abstract

The enhanced permeability and retention (EPR) effect has become the guiding principle for nanomedicine against cancer for a long time. However, several biological barriers severely resist therapeutic agents’ penetration and retention into the deep tumor tissues, resulting in poor EPR effect and high tumor mortality. Inspired by lava, we proposed a proteolytic enzyme therapy to improve the tumor distribution and penetration of nanomedicine. A trypsin-crosslinked hydrogel (Trypsin@PSA Gel) was developed to maintain trypsin’s activity. The hydrogel postponed trypsin’s self-degradation and sustained the release. Trypsin promoted the cellular uptake of nanoformulations in breast cancer cells, enhanced the penetration through endothelial cells, and degraded total and membrane proteins. Proteomic analysis reveals that trypsin affected ECM components and down-regulated multiple pathways associated with cancer progression. Intratumoral injection of Trypsin@PSA Gel significantly increased the distribution of liposomes in tumors and reduced tumor vasculature. Combination treatment with intravenous injection of gambogic acid-loaded liposomes and intratumoral injection of Trypsin@PSA Gel inhibited tumor growth. The current study provides one of the first investigations into the enhanced tumor distribution of liposomes induced by a novel proteolytic enzyme therapy.

**Graphic Abstract:**

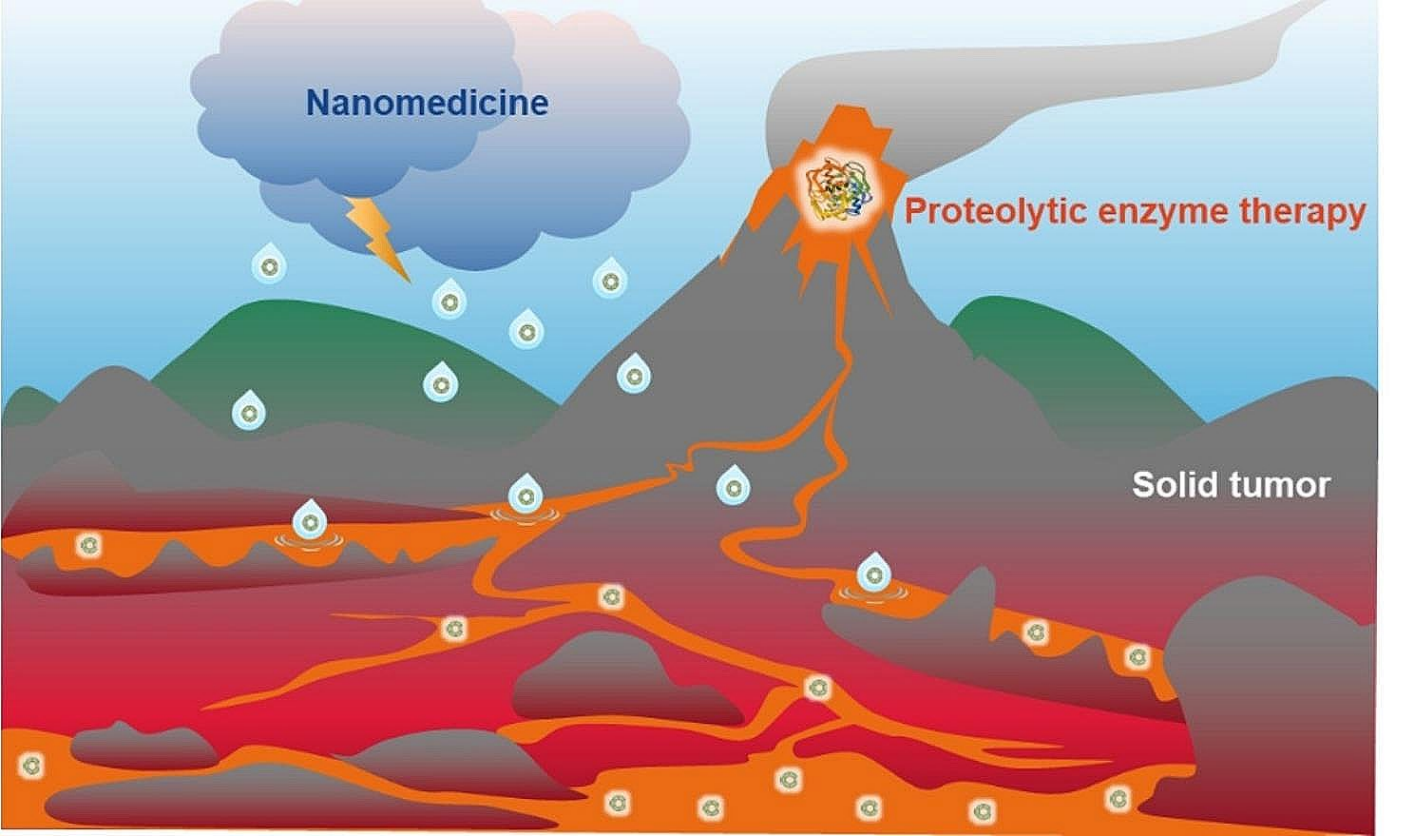

**Supplementary Information:**

The online version contains supplementary material available at 10.1186/s12951-024-02468-7.

## Introduction

Tumor targeting by drug delivery systems (DDS) has been well explored to overcome the shortcomings of conventional chemotherapy, such as poor solubility, limited bioavailability, and a few side effects [1–4]. The major underlying mechanism of DDS to fight against cancer is the Enhanced Permeability and Retention (EPR) effect [5]. The EPR effect refers to the concept that the increased permeability of nanoparticles has been attributed to large inter-endothelial gaps and poor lymphatic drainage. Since the first report of the EPR effect, it has been considered the “royal gate” in the drug delivery field since drug-loaded nanomedicine could accumulate inside a tumor and reduce the side effect associated with systematic chemotherapy [6–9]. The EPR effect has led to a boom in research on DDS and the production of many DDS formulations. Some of them have also been applied in clinical trials. However, although the EPR effect of DDS has been investigated worldwide in publications with animal experiments, it has not worked well in clinical practice [10]. For instance, doxorubicin-loaded liposomes with an expectation of both passive and active targeting failed to reveal any positive responses in patients with gastric cancer [11]. One of the major differences between xenografts and clinical cancer is the abundance of cancer stroma. Clinical cancer tissues often contain abundant cancer stroma, whereas xenograft does not have it [10]. The dense stroma is composed of specialized connective tissues and the extracellular matrix (ECM), which is responsible for inhibiting the penetration of nanomedicines [12–14]. Researchers have developed a few strategies to accelerate the EPR effect of nanoparticles in deep tumor tissue. One of the novel approaches is the degradation of collagen, the most abundant components of the ECM, by collagenase-conjugated nanoparticles [14, 15]. However, tightly packed tumor cells [16] and limited inter-endothelial gaps in the tumor blood vessel [17] are still obstacles to the deep penetration of nanoparticles. Indeed, Chen and colleagues analyzed more than 300 blood vessels across three breast cancer models and observed 26 gaps by transmission electron microscopy. The overall gap coverage was only 0.048% of the vascular surface area [17]. Hence, a strategy degrades the dense ECM, connected tumor cells, and tight junctions between vascular endothelial cells might be helpful to improve the distribution of nanomedicines in tumor tissue.

Trypsin (Fig. S1), an endogenous serine protease that cleaves explicitly between lysine and arginine residues, is commonly used to detach adherent cells in cell culture. Thus, we hypothesize that trypsin may digest the dense tumor stroma, compact tumor cells, and tight junctions between endothelial cells, leading to an enhanced tumor distribution of nanomedicine. Evidence has supported the antitumor efficacy of enzyme therapy [18–20]. Clinical studies of systemic delivering proteolytic enzymes against oncology combined with chemotherapy or radiotherapy resulted in reduced adverse effects and even prolonged survival [21]. It was mentioned that trypsin reduced cancer stem cells’ population and impaired their metastatic capacity and the epithelial-to-mesenchymal (EMT) transition [18]. Besides, its mechanism of action against cancer remains unclear, with few articles published. In this study, inspired by the phenomenon that lava erosion accelerates rainwater penetration into the stone mountain, we assume trypsin might be a potent anticancer agent that synergizes with nanomedicine.

However, trypsin is essentially a protein that undergoes the process of self-digestion and loses its activity in physical conditions [22]. Various delivery carriers with desired functions, such as lipid nanocapsules, polymeric nanoparticles, and inorganic nanoparticles, were developed to enhance the biopharmaceutical applications of proteins [23–25]. The entrapment of proteins in these carriers often requires laborious optimization, harsh conditions involving organic solvents, and frequently results in poor encapsulation efficiencies [26]. Direct injection of protein systemically has proven to have limited clinical success due to short half-lives and systemic off-target effects. Hydrogels are widely applied in the field of materials and biomedicine [27–31] and are suitable carriers to locally confine bioactive guests, such as enzymes. Nevertheless, the preparation techniques of hydrogels may affect the final performance of the immobilized enzyme; the organic chemical processes or radical initiators usually damage their active structures [32]. To minimize trypsin’s self-degradation and maintain its activity, we prepared a trypsin-crosslinked hydrogel containing PEG-SH and AgNO_3_ (Trypsin@PSA Gel). Trypsin was an assisted crosslinker and formed Ag-S bonds with Ag^+^, promoting the gelation process without crosslinking agents that inhibit its activity. The hydrogel protected trypsin from degradation and sustained its release at physiological and tumor microenvironmental pH.

**Scheme 1:**
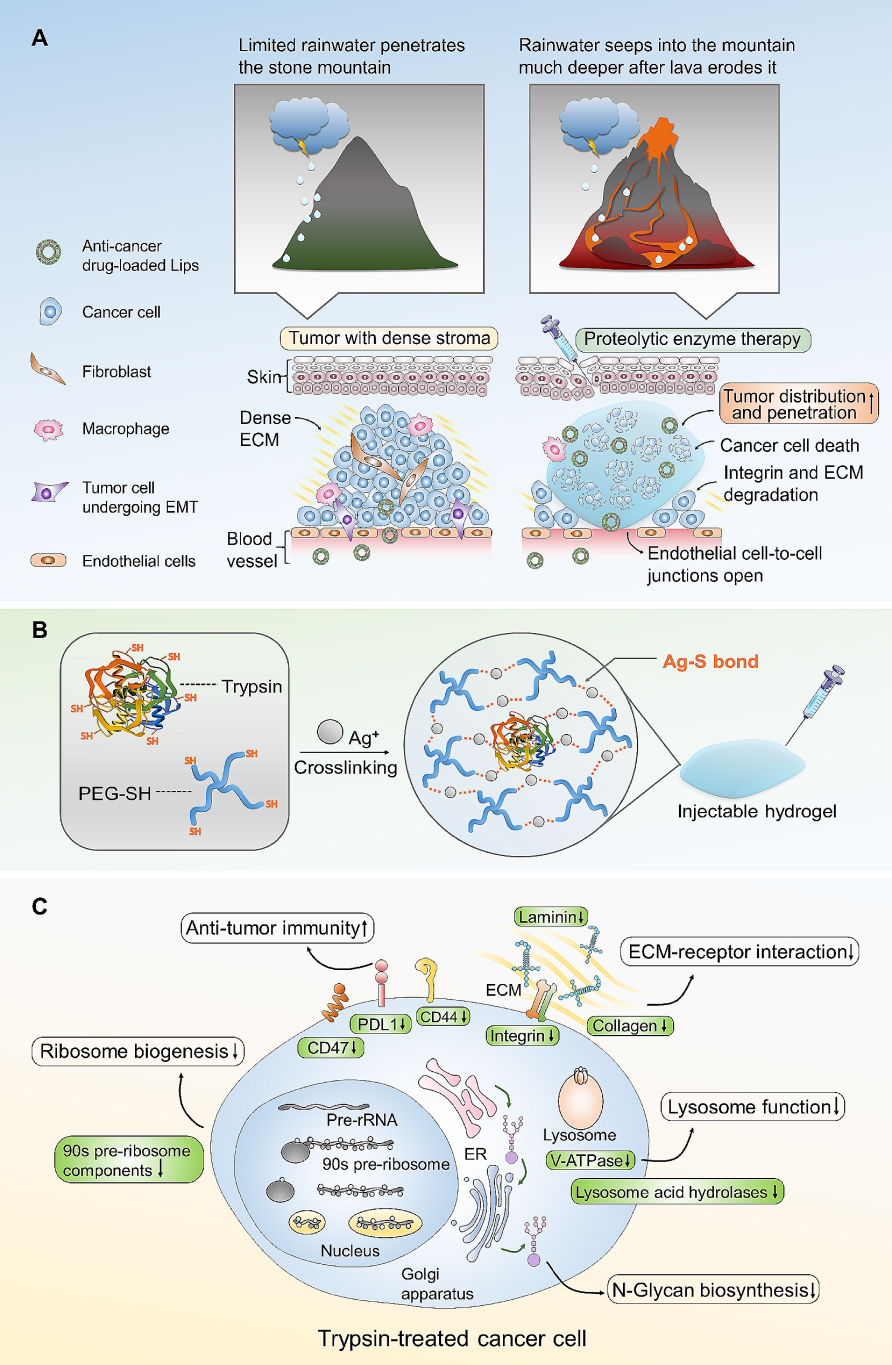
Schematic illustration of Trypsin@PSA Gel-mediated proteolytic enzyme therapy. (**A**) Lava-inspired proteolytic enzyme therapy with Trypsin@PSA Gel improved tumor distribution and penetration of liposomes. (**B**) Formation of Trypsin@PSA Gel. Trypsin facilitates the gelation of PEG-SH and AgNO_3_ based on Ag-S bonds. (**C**) Overall effects of trypsin on tumor cells and tissues include down-regulating ECM-receptor interaction, lysosome function, N-glycan biosynthesis, ribosome biogenesis, and improving antitumor immunity

In the current work, a trypsin-based therapy was applied to enhance the tumor distribution of Gambogic acid (GA) nanomedicine, a xanthone isolated from the exudates of *Garcinia hanburyi* which was demonstrated to be efficient for TNBC treatment [31]. We evaluated the effect of trypsin on cellular uptake of three nanoformulations applied in systemic drug delivery, including liposomes (Lip), poly (lactic-co-glycolic acid) nanoparticles (PLGA NP), and bovine serum albumin nanoparticles (BSA NP). An in vitro tight junction model was developed to study the penetration of those nanoformulations loaded with GA through endothelial cells pre-treated with trypsin. We performed a label-free quantitative proteomic analysis to reveal important alterations in the proteome of trypsin-treated tumor cells. A breast cancer cell line 4T1 was selected for in vitro and in vivo analysis since the 4T1 syngeneic model presents a dense stroma relevant to human tumors [17]. The distribution of Lips after intratumoral injection of Trypsin@PSA Gel was evaluated in 4T1 tumor-bearing mice. The antitumor efficacy of combination treatment with GA-loaded Lips and the Trypsin@PSA Gel was also evaluated. We hope this trypsin-crosslinked hydrogel will contribute to the distribution and penetration of nanomedicine in tumor and overcome the hurdles in the EPR effect (Scheme 1).

## Materials and methods

### Materials

GA was bought from PureChem (China). PLGA (RESOMER® RG 502 H) was obtained from Evonik (China). Fluorescein sodium salt was purchased from Sigma (USA). BSA and trypsin (Mw, ∼ 23.8 kDa; activity, ≥ 250 U/mg) were purchased from Biofroxx (Germany). DAPI and DiD were obtained from Bioscience Technology (China). Transwell® (1 μm pore size) was purchased from Jet Bio-Filtration (China). Gelatin (250 g Bloom) was supplied by Macklin Biochemical (China). AgNO_3_, phosphatidylcholine (PC), cholesterol, ethanol absolute, dichloromethane, trichloromethane, dimethyl sulfoxide (DMSO), and other chemical reagents were purchased from Kelong Chemical (China) .

RIPA, PMSF, and phosphatase inhibitor cocktail were bought from Servicebio (China). β-tublin and PD-L1 antibodies were acquired from Abmar (USA). CD31 and CD44 antibodies were purchased from ImmunoWay (USA). YF®488-conjugated goat anti-rabbit IgG was obtained from US Everbright (China). FITC-conjugated anti-CD44 was bought from 4 A Biotech (China).

### Cell culture

Dulbecco’s modified Eagle’s medium (DMEM) and Roswell Park Memorial Institute (RPMI) 1640 medium were purchased from Corning (USA). Fetal bovine serum (FBS) was purchased from Excell Bio (China). The mouse cell lines 4T1, bEnd.3, and 4T1-Luc were obtained from ATCC. Cells were cultured in DMEM or RPMI 1640 medium containing 1% antibiotics (penicillin-streptomycin, 10,000 U/mL) and 10% FBS in an atmosphere of 5% CO_2_ at 37 °C.

### Structure of trypsin

Trypsin’s crystal structure was acquired from the Protein Data Bank (PDB ID: 1MCT) and depicted with rainbow ribbons on a white background. The pink stick-and-surface model reveals the structure of cysteines (Cys), while the orange ball model reveals the structure of calcium ion.

### Preparation and characterization of GA nanoformulations

GA-loaded Lip, BSA NP, and PLGA NP were prepared based previously reported method [33–35]. To prepare GA-loaded Lip, PC, cholesterol, and GA were dissolved in trichloromethane, and the solution was rotary evaporated to form a thin film, followed by adding PBS to hydrate. The sonication was performed in an ice-water bath for 10 min to obtain GA-Lip. To prepare GA-BSA NP, GA was dissolved in the dichloromethane and ethanol, then added to BSA solution, sonicated, and rotated evaporation to remove the organic solvent. To prepare GA-PLGA NP, PLGA and GA were dissolved in dichloromethane, and then added to 3% (w/v) PVA solution, emulsified using a vortex and sonicated. The mixture was rotary evaporated until the organic solvent was removed. The suspension was filtered and washed by centrifugation.

The particle size, polydispersity index (PDI), and zeta potential of these formulations were diluted with RO water and measured by a Litesizer™ 500 (Anton Paar, Austria). Drug loading and encapsulation efficiency of GA were detected using high-performance liquid chromatography (HPLC) (Shimadzu, Japan) with the chromatographic condition of 90% (*v/v*) acetonitrile and 10% (*v/v)* 0.2% phosphoric acid solution.

### Internalization of nanoformulations after trypsin treatment

Nanoformulations were labeled with Coumarin 6 (C6) to measure the effect of trypsin on cell uptake and internalization. 4T1 breast cancer cells were seeded in 12-well plate at a density of 2 × 10^5^ cells/well and incubated for 24 h. After treatment with trypsin for 2 min, cells were incubated with C6 nanoformulations (2 µg/mL C6) for 2 h and collected. Cells were transferred to climbing slices, fixed after 24 h’s recovery, and stained by DAPI. The internalizations of C6 nanoformulations were observed through an FV1200 scanning confocal microscope (Olympus, Japan). Cell samples for flow cytometry were washed, resuspended in phosphate-buffered saline (PBS), and analyzed through FACSVerse™ (BD, USA).

To investigate trypsin’s effect on cell membranes, 4T1 cells were collected after treatment with trypsin for 2 min and 30 min. Cell membranes and nucleus were stained with DiD and DAPI for visualization using confocal microscopy. Meanwhile, cells were treated with 0.5% trypsin for 30 min, washed with PBS, and fixed by electron microscope fixative at 4 ℃ followed by SEM observation.

### Measurement of total and membrane proteins after trypsin treatment

The 4T1 cells were seeded in 6-well plate at a density of 1 × 10^6^ cells/well and incubated for 24 h. After treatment with different concentrations (0.125-1%, *w/v*) of trypsin, 4T1 cells were washed with PBS and lysed in RIPA buffer containing PMSF and phosphatase inhibitor cocktail to extract the total proteins. The membrane proteins were extracted by a membrane extraction kit (Sangon Biotech, China). Total and membrane proteins with or without trypsin treatment were measured by the Bradford protein determination assay, followed by separation with SDS-PAGE gel. The obtained gel was stained with the Coomassie Blue superfast staining solution (Beyotime) for gel imaging. To detect CD44 and PD-L1 expression on trypsin-treated 4T1 cells, a western blot and flow cytometry analysis was performed. For western blot, total proteins were transferred onto the PVDF membrane. The membranes were soaked in a blocking solution, followed by incubation with the primary antibodies at 4 ℃ and the secondary antibody at room temperature. CD44 and PD-L1 were visualized by an OI 600 automatic chemiluminescence imaging system (BIO-OI, China). Tumor cells for flow cytometry analysis were treated with FITC-conjugated anti-CD44 or primary antibody against PD-L1, followed by incubating with YF^®^ 488-conjugated goat anti-rabbit IgG secondary antibody.

### Endothelial permeability of nanoformulations after trypsin treatment

To evaluate the permeability of nanoformulations through the tight junction of endothelial cells, we developed an in vitro tight junction model with the immortalized mouse brain endothelial cell line, bEnd3, in a Transwell® system. bEnd.3 cells were cultured on Transwell® membranes (24-well type) coated with 2% gelatin for 5 d to form a confluent cell layer at an initial density of 3 × 10^4^ cells/well. A 4 h leaking test and fluorescein sodium permeability assay confirmed the tight junction formation [36, 37]. The in vitro tight junction model was treated with trypsin-containing serum-free medium for 30 min. Trypsin was removed and washed with PBS. C6 nanoformulations were added to the endothelial layer made by bEnd.3 cells. At the indicated time point (0 min, 10 min, 30 min, and 60 min), 100 µL of media in the lower chamber was collected and replenished with an equal amount of culture medium. The permeability rate of C6 nanoformulations was calculated by measuring the fluorescence intensity of C6 in the collected medium using a SpectraMax® i3 microplate reader (Molecular Devices, USA).

### In vitro cell viability and apoptosis analysis

The viability of 4T1 cells treated with GA and GA nanoformulations was assessed by an MTT assay. 4T1 cells were seeded in a 96-well plate at a density of 5 × 10^3^ cells/well and incubated with free GA or GA nanoformulations for 48 h. The MTT solution subsequently replaced the culture medium. The MTT solution was discarded after 4 h’s incubation. Then, the formazan crystals were dissolved by adding DMSO. The absorbance of the formazan crystals was measured at 490 nm using a SpectraMax® i3 microplate reader. The viability of 4T1 cells treated with trypsin was assessed by an CCK-8 assay with the same procedure mentioned above. Each well was added with 10 µL of CCK-8 solution and incubated for 2 h. Formazan was measured at 450 nm by the microplate reader.

Apoptosis of 4T1 cells was analyzed after sequential treatment with trypsin and GA nanoformulations in the Transwell® system mentioned above. bEnd.3 cells in a Transwell® system were treated with different concentrations (0.125-1%, *w/v*) of trypsin in a serum-free medium for 30 min. The Transwell® insert was moved to a 24-well plate with 4T1 cells grown on the lower chamber at an initial density of 3 × 10^5^ cells/well. The whole system was treated with GA nanoformulations for 1 h, followed by the removal of the upper Transwell®. Finally, 4T1 cells were collected after incubation with GA nanoformulations for another 48 h. The cells were stained using Annexin V-fluorescein isothiocyanate (Annexin-V-FITC)/propidium iodide (PI) kit and analyzed by flow cytometry.

The viability of 4T1 cells was tested after concurrent treatment with trypsin and GA nanoformulations on 4T1 cells and bEnd.3 co-cultured model. The model was established by 4T1 cells seeded to a 24-well plate with bEnd.3 culturing on the upper Transwell®. The whole system was treated with GA nanoformulations and trypsin for 48 h. The MTT assay was performed to measure the viability of 4T1 cells. The absorbance was measured at 570 nm on a microplate reader.

### Proteomics analysis

The label-free quantitative proteomic assessment was conducted by PTM BIO (China). In brief, 4T1 cells post-treatment with 0.5% trypsin (0.2 µmol/L) for 30 min were sonicated three times on ice using a high-intensity ultrasonic processor (Scientz, China) in lysis buffer (8 mol/L urea, 1% protease inhibitor cocktail). The remaining debris was removed by centrifugation. The supernatant was collected, and the protein concentration was calculated with a BCA kit according to the manufacturer’s instructions. The protein solution was reduced with 5 mmol/L dithiothreitol for 30 min at 56 °C and alkylated with 11 mM iodoacetamide for 15 min at room temperature in darkness. The protein sample was diluted by adding 100 mmol/L TEAB to a urea concentration of less than 2 mol/L. Finally, trypsin was added at a 1:50 trypsin-to-protein mass ratio for overnight digestion.

The tryptic peptides were dissolved in solvent A (0.1% formic acid, 2% acetonitrile/in water) and directly loaded onto a homemade reversed-phase analytical column. Peptides were separated with a nanoElute^®^ UHPLC system (Bruker Daltonics). The peptides were subjected to a capillary source followed by the timsTOF Pro (Bruker Daltonics) mass spectrometry. The electrospray voltage applied was 1.60 kV. Precursors and fragments were analyzed at the TOF detector, with an MS/MS scan range from 100 to 1700 m/z. The timsTOF Pro was operated in parallel accumulation serial fragmentation (PASEF) mode. Precursors with charge states 0 to 5 were selected for fragmentation, and 10 PASEF-MS/MS scans were acquired per cycle. The dynamic exclusion was set to 30 s.

### Construction and characterization of hydrogels

Trypsin@PSA Gel was prepared by mixing 4-arm PEG-SH (20 kDa, Yare Biotech, China), AgNO_3_, and trypsin. Briefly, 20 mg of 4-arm PEG-SH was weighted and dissolved in 100 µL of 45 mg/mL trypsin in deionized water (solution A). The solution A was mixed with 200 µL of AgNO_3_ solution in different concentrations. Unloaded PSA was prepared by replacing trypsin with deionized water.

Trypsin@PSA Gel with trypan blue was injected into water through a 26 G needle to investigate its injectability. PSA and Trypsin@PSA Gel were freeze-dried to observe the morphology by scanning electron microscope (SEM) (Zeiss, Germany). The viscoelasticity of PSA and Trypsin@PSA Gel was measured by a MARS™ rheometer (Thermo Fisher, USA). The storage modulus (G′) and loss modulus (G″) were assessed at 6.28 rad/s, with the shear strain amplitude ranges from 0.1–100%. Raman spectra were measured by an Alpha300R Raman spectrometer (WiTech, Germany) using an argon laser with a wavelength of 488 nm as the excitation source. The swelling properties of hydrogels were studied by the gravimetric method. Trypsin@PSA Gel and PSA were weighed before and after the immersion with PBS for 3 d.

### Activity, stability, and release profiles of trypsin in Trypsin@PSA Gel

Trypsin activity in Trypsin@PSA Gel was measured by a trypsin activity assay kit (Jining, China) at pH 7.4. The Bradford protein assay kit (Beyotime, China) was used to investigate trypsin’s stability and release profiles in Trypsin@PSA Gel at pH 6.5 and 7.4. Before measuring the activity and stability, Trypsin@PSA Gel were diluted in water to make the solid hydrogel into a colloidal sol. To measure the release profile of trypsin, Trypsin@PSA Gel was freeze-dried and added to 5 mL media at pH 6.5 and 7.4. Samples were shaken at 37 ℃ at 100 rpm/min. At certain time points, 50 µL of the release medium was collected to measure the amount of released trypsin.

### Biocompatibility of unloaded PSA

BALB/c mice were randomly divided into three groups to explore the biocompatibility of the hydrogel. Mice were injected subcutaneously with 100 µL of PBS or PSA and sacrificed on day 7. Tissue around the injection site was collected and fixed with 4% formaldehyde for H&E staining.

### Biodistribution of DiD-labeled lip after intratumoral injection of Trypsin@PSA Gel

The animal experiments were approved by the ethics committee of the Chengdu University of Traditional Chinese Medicine (2022-45), and all animal experiments were conducted in strict accordance with the Guidelines for the Care and Use of Laboratory Animals of the Ministry of Science and Technology of China. The subcutaneous breast cancer model was established by injecting 1 × 10^6^ 4T1 cells into the lower right abdomen of female BALB/c mice (18–20 g). DiD-labeled Lip was prepared for in vivo visualization. When tumor volume reached 200–300 mm^3^, mice were randomly divided into five groups (*n* = 3): Blank, DiD-Lip, PSA + DiD-Lip, trypsin + DiD-Lip, and Trypsin@PSA Gel + DiD-Lip. DiD-Lip was injected 24 h after intratumoral injection of PBS, PSA, trypsin, or Trypsin@PSA Gel. The administrated doses included: DiD (0.2 mg/kg) and trypsin (0.3 mg per tumor). At 4 h, 8 h, and 24 h after DiD-Lip injection, mice were anesthetized with isoflurane and photographed by an IVIS^®^ spectrum imaging system (PerkinElmer, USA). Then, mice were sacrificed at 48 h. Tumors were collected, photographed, and placed at -80 ℃. For immunofluorescence staining, tumors were treated with primary antibodies against CD31 at 37 °C, washed three times with PBS, and incubated with YF^®^ 488-conjugated goat anti-rabbit IgG for 30 min.

### In vivo antitumor efficacy

The subcutaneous breast cancer model was developed by injecting 3 × 10^6^ 4T1-Luc cells into the right flank of BALB/c mice. One week later, mice were randomly divided into four groups (*n* = 8): PBS, GA-Lip, Trypsin@PSA Gel, and Trypsin@PSA Gel + GA-Lip. Specifically, PBS or Trypsin@PSA Gel were injected intratumorally on days 1, 3, 5, 7, and 9. GA-Lip was injected intravenously on days 2, 4, 6, 8, and 10. The administrated doses included: GA (12 mg/kg) and trypsin (0.3 mg per tumor). The body weight and tumor volume of treated mice were monitored every other day. On day 16, all mice were sacrificed, and the tumor weight was measured. Tumors were fixed with 4% formaldehyde for H&E, TUNEL, and immunofluorescence-stained with FITC-conjugated anti-CD44. SEM images of tumor obtained from PBS and the Trypsin@PSA + GA-Lip group were captured to illustrate the tumor vasculature.

## Results and discussion

### Trypsin treatment yields higher internalization of nanoformulations

**Fig. 1:**
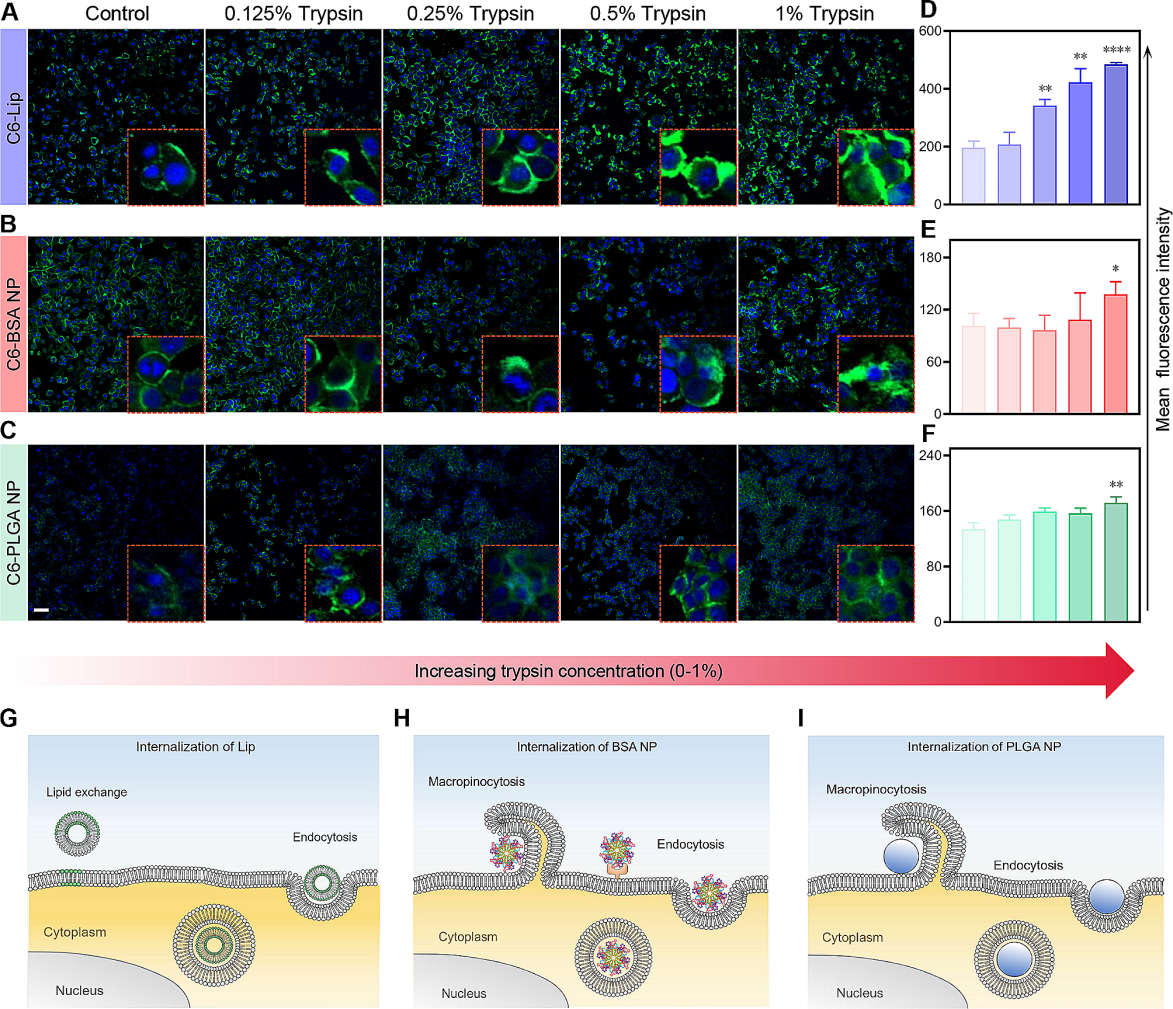
Influence of short-time trypsin treatment on internalization of C6-labeled nanoformulations. (**A**-**F**) Representative confocal images (**A**-**C**) and flow cytometry results (**D**-**F**) of different nanoformulations’ cellular uptake by 4T1 cells, scale bar = 50 μm. (**G**-**I**) Schematic illustration showing the cellular uptake mechanisms of different nanoformulations. All statistical data are presented as mean ± SD, *n* = 3. *p < 0.05, **p < 0.01, ****p < 0.0001

Nanomedicines enter cells in various ways, such as endocytosis and receptor-mediated endocytosis. Trypsin is a proteinase for digesting cell clusters. It enables the degradation of ECM and increases the contact area with nanomedicine, impacting nanomedicines’ internalization. Then, we prepared Lip, BSA NP, and PLGA NP to evaluate such influences (Table S1; Fig. S2-S4). Figure 1A-C shows fluorescence images of the 4T1 cells exposed to different coumarin-6 (C6)-labeled nanoformulations after a 2-min treatment with trypsin in different concentrations. Few nanoformulations were uptook by 4T1 tumor cells without trypsin treatment. In contrast, the internalization of all nanoformulations was substantially higher by increasing the concentration of trypsin. Results from flow cytometry analysis indicate a similar trend in Fig. 1D-F. The intensity of fluorescence varies among three nanoformulations. A possible explanation might be that trypsin treatment degrades part of the ECM and membrane proteins, making cells detachable from the culture plate. The internalization of nanoformulations might benefit from the increased contact area and reduced ECM interference. The different fluorescence intensities detected could be explained by different mechanisms of internalization (Fig. 1G-I). Lips interact with the cell through multiple mechanisms, such as lipid exchange and endocytosis [38]. The internalization of PLGA and BSA NP mainly depends on clathrin- or caveolae-mediated endocytosis and macropinocytosis [39–41]. The efficiency of endocytosis is ATP-dependent. Nevertheless, soft Lips deform to increase their contact area with the cell membrane [38]; therefore are more likely to be affected by contact area with cells.

### Trypsin treatment permeabilizes cellular membranes by degrading total and membrane proteins

**Fig. 2:**
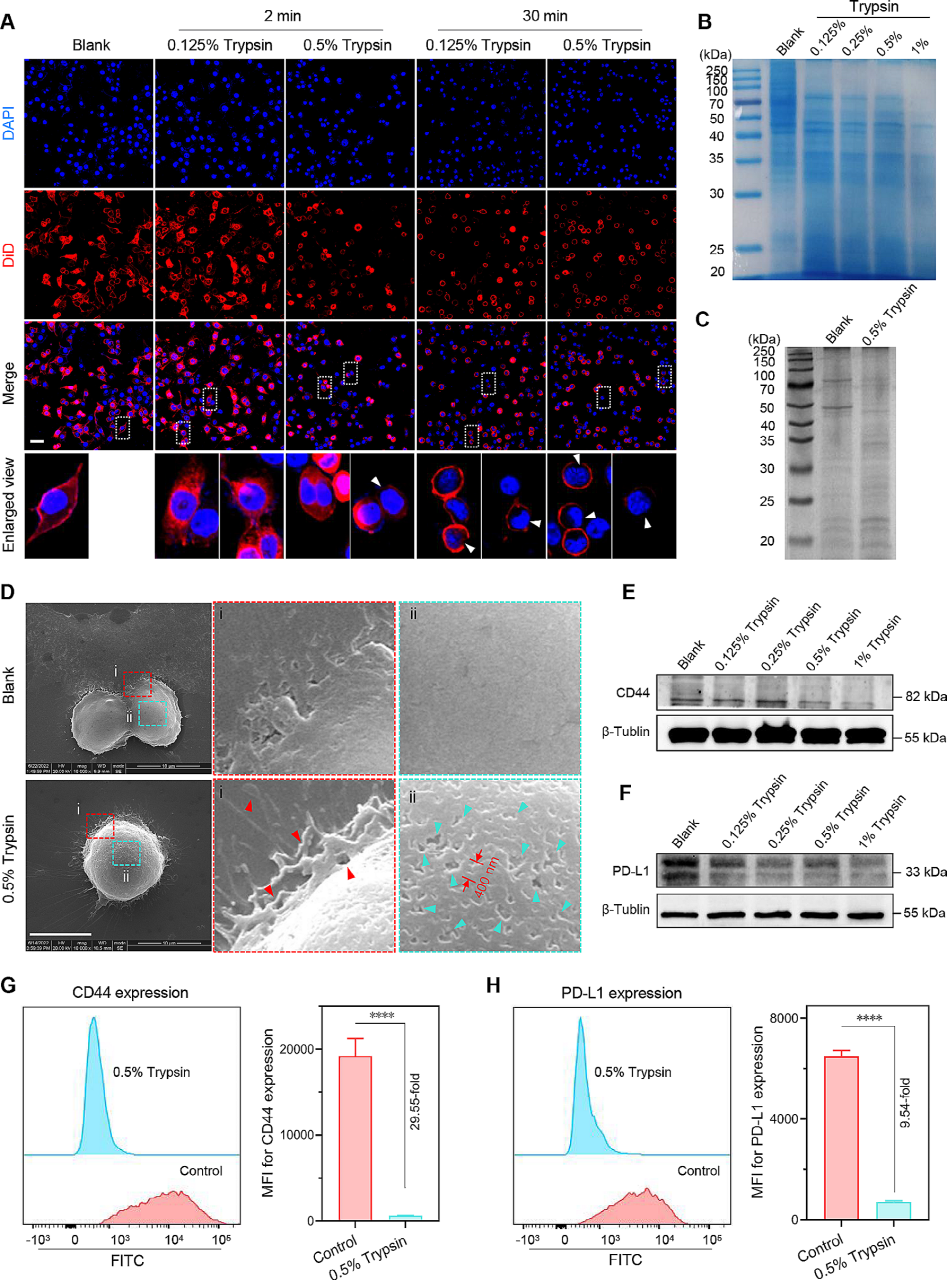
Trypsin treatment permeabilizes 4T1 cell membranes by degradation of total and membrane proteins. (**A**) DiD-labeled cell membranes after trypsin treatment, scale bar = 50 μm (the white arrows indicate the damaged membrane). (**B**) Total protein levels after incubating with trypsin for 30 min. (**C**) Membrane protein levels after 0.5% trypsin treatment for 30 min. (**D**) Representative SEM images of cell membranes with 0.5% trypsin incubating for 30 min, scale bar = 10 μm (the red and cyan arrows indicate the damages on the surface of 4T1 cells). (**E**-**F**) Western blot analysis of (**E**) CD44 and (**F**) PD-L1 expression in total proteins after 0.5% trypsin digestion for 30 min. (**G**-**H**) Flow cytometry results of (**G**) CD44 and (**H**) PD-L1 positive cells after 0.5% trypsin digestion for 30 min. All statistical data are presented as mean ± SD, *n* = 3. ****p < 0.0001

To find out the effect of trypsin treatment on cell membranes, 4T1 cells were stained with a lipophilic fluorescent DiD to identify the cell membrane. As shown in Fig. 2A, the signal of DiD was significantly reduced after treatment with a higher concentration of trypsin for a longer time, indicating the damages of cell membrane. Then, total proteins were extracted from trypsin-treated 4T1 tumor cells to confirm this conjecture. Sodium dodecyl sulfate (SDS)-PAGE gel reveals a gradual drop in protein abundance after treatment with incremental concentrations of trypsin for 30 min (Fig. 2B and Fig. S5A). Then, the same technique was performed to determine whether trypsin treatment affects membrane proteins. Figure 2C and Fig. S5B indicates a noticeable decline in membrane protein abundance after 0.5% trypsin digestion for 30 min. Then, SEM images were recorded to obtain information on cell membrane integrity. What can be seen in Fig. 2D is the phenomenal damage to the cell membrane by 0.5% trypsin treatment for 30 min, with countless tiny pores (∼ 400 nm) on the cell membrane. These tiny pores might also be helpful for the penetration of nanomedicines through cell membranes. These findings help us understand trypsin’s digestive ability and influences on cell membranes. To investigate if antitumor efficacy can be beneficial from trypsin treatment. We performed western blot and flow cytometry analysis on two proteins associated with antitumor immunity and cancer stemness. There was a gradual decline in the levels of CD44 or PD-L1 by increasing the concentration of trypsin (Fig. 2E, F and Fig. S6). Flow cytometry analysis also reveals a decrease of CD44 or PD-L1 positive cells as expected after 0.5% trypsin treatment (Fig. 2G and H). In summary, short-time trypsin treatment facilitated the internalization of nanoformulations. However, long-time trypsin treatment for over 30 min permeabilized cellular membranes and damaged total and membrane proteins.

### Trypsin treatment enhances nanoformulations’ penetration through endothelial cells

**Fig. 3:**
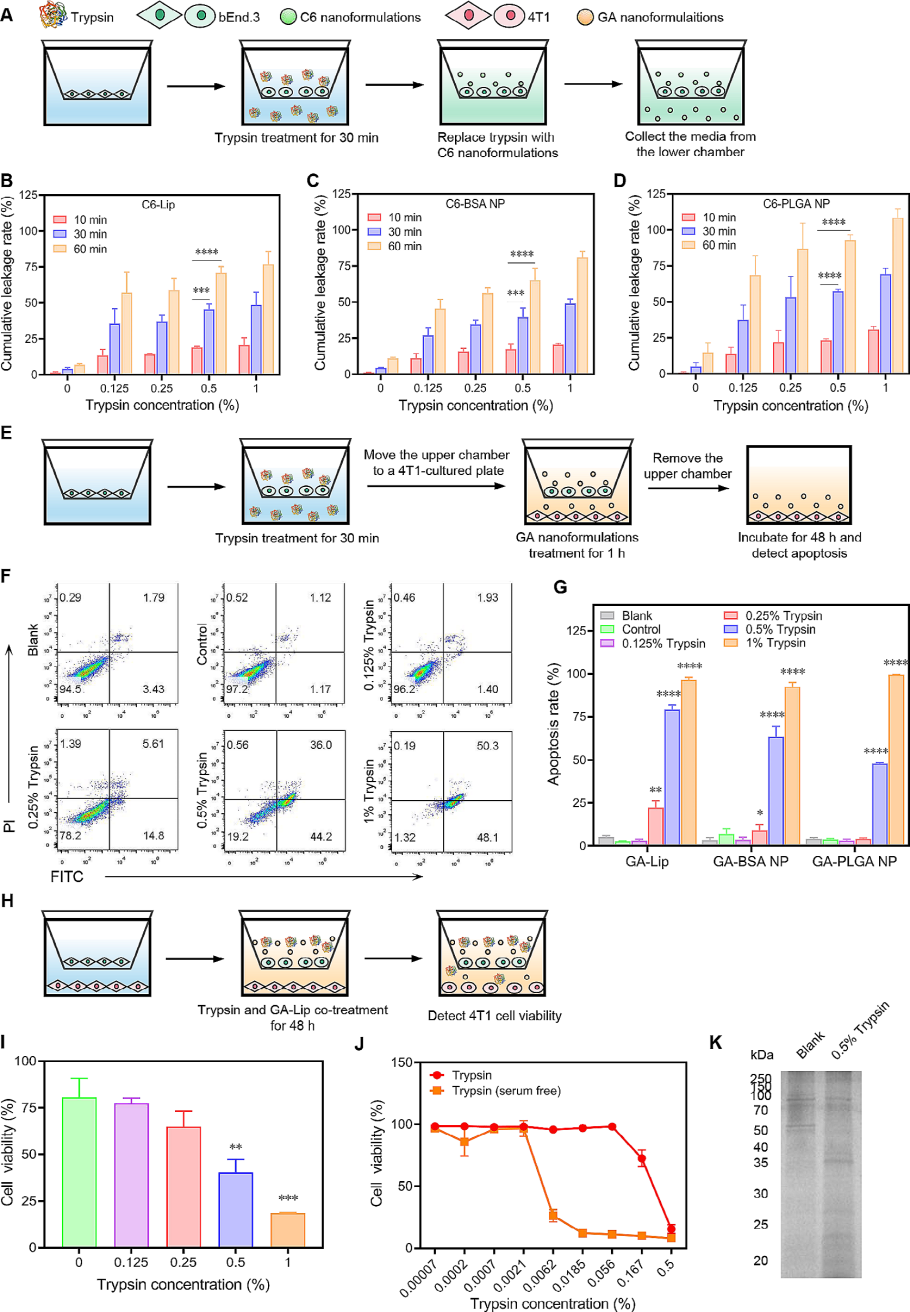
Trypsin treatment enhances nanoformulations’ penetration through endothelial cells. (**A**) Schematic illustration of (**B**)-(**D**). (**B**-**D**) Cumulative leakage rate of different nanoformulations through bEnd.3 cells pre-treated with trypsin for 30 min in a Transwell® system. (**E**) Schematic illustration of (**F**) and (**G**). (**F**-**G**) Flow cytometry results of apoptosis after trypsin pre-treatment followed by GA nanoformulations administration in a Transwell® system. (**H**) Schematic illustration of (**I**). (**I**) Cell viability of 4T1 after trypsin and GA-Lip co-treated for 48 h in a Transwell® system. (**J**) Cell viability of 4T1 with trypsin treatment for 48 h. (**K**) SDS-PAGE results indicating membrane protein levels of bEnd.3 cells after 0.5% trypsin treatment for 30 min. All statistical data are presented as mean ± SD, *n* = 3. **p < 0.01, ***p < 0.001, ****p < 0.0001

After exploring the impact of trypsin on cell membrane, we were motivated to study its influences on nanoformulations’ penetration through endothelial cells. We developed an in vitro endothelial cell tight junction model with a Transwell® system. Briefly, bEnd.3 cells were laid on a Transwell® plate and cultivated for a few days to develop the tight junction model. After confirming the tight junction formation (Fig. S7), bEnd.3 cells were incubated with trypsin for 30 min, followed by the removal of it (Fig. 3A). Then, C6-labeled Lip, PLGA NP, and BSA NP were added to the upper chamber for incubation. The cumulative leakages of those nanoformulations through tight junctions were evaluated by fluorescence intensity test. As indicated in Fig. 3B-D, the detected fluorescence intensities increased by enhancing trypsin concentrations and the incubation time. Such a result might be explained by the destroyed tight junctions between endothelial cells. Indeed, SDS-PAGE gel shows a notable drop in membrane protein abundance of bEnd.3 after treatment with 0.5% trypsin (Fig. 3K).

Further, the trypsin-treated bEnd.3 cells in Transwell® inserts were moved to a new 24-well plate with 4T1 cells seeded at the bottom chamber. GA-loaded Lip, PLGA NP, and BSA NP were added to the upper chamber and incubated for 1 h (Fig. 3E). An Annexin V-fluorescein isothiocyanate (Annexin-V-FITC)/propidium iodide (PI) kit was used to detect the apoptosis of 4T1 cells induced by the penetrated GA nanoformulations. As shown in Fig. 3F, G and Fig. S8-S9, treatment of bEnd.3 in the upper chamber with 0.5% and 1% trypsin notably enhanced the apoptosis rate of 4T1 cells in the bottom well. The breakdown of tight junctions by high concentrations of trypsin leads to the leakage of drug-loaded nanoparticles to interact with 4T1 tumor cells. The transwell system was co-incubated with trypsin and GA-Lip for 48 h (Fig. 3H). Previous treatments indicate that most of the GA-Lips were penetrated to the bottom through the bEnd.3 tight junctions in this condition. Increasing the concentration of trypsin led to a continued decrease in cell viability without changing the amount of GA-Lip (Fig. 3I). Although GA or GA nanoformulations inhibit tumor cell viability (Fig. S10), trypsin promotes the leakage of nanoparticles and directly inhibits tumor cell growth. A CCK-8 assay was performed directly on 4T1 cells to investigate the effect of trypsin alone on tumor cell viability (Fig. 3J). The cell viability curve treated with trypsin was drastically different in fetal bovine serum (FBS)-containing and FBS-free conditions. The IC_50_ value of trypsin is 0.27% and 0.00535% in FBS-containing and FBS-free conditions, respectively. The serum is known to inactivate trypsin via protease inhibitors, such as α1-antitrypsin [42]. Direct injection of trypsin into the tumor might cause the inactivation of trypsin. It highlights the critical role of hydrogel in protecting trypsin from contact with the nutrient-rich tumor microenvironment and facilitating local active concentration.

### Label-free quantitative proteomics reveals important alterations in the proteome of trypsin-treated 4T1 cells

**Fig. 4:**
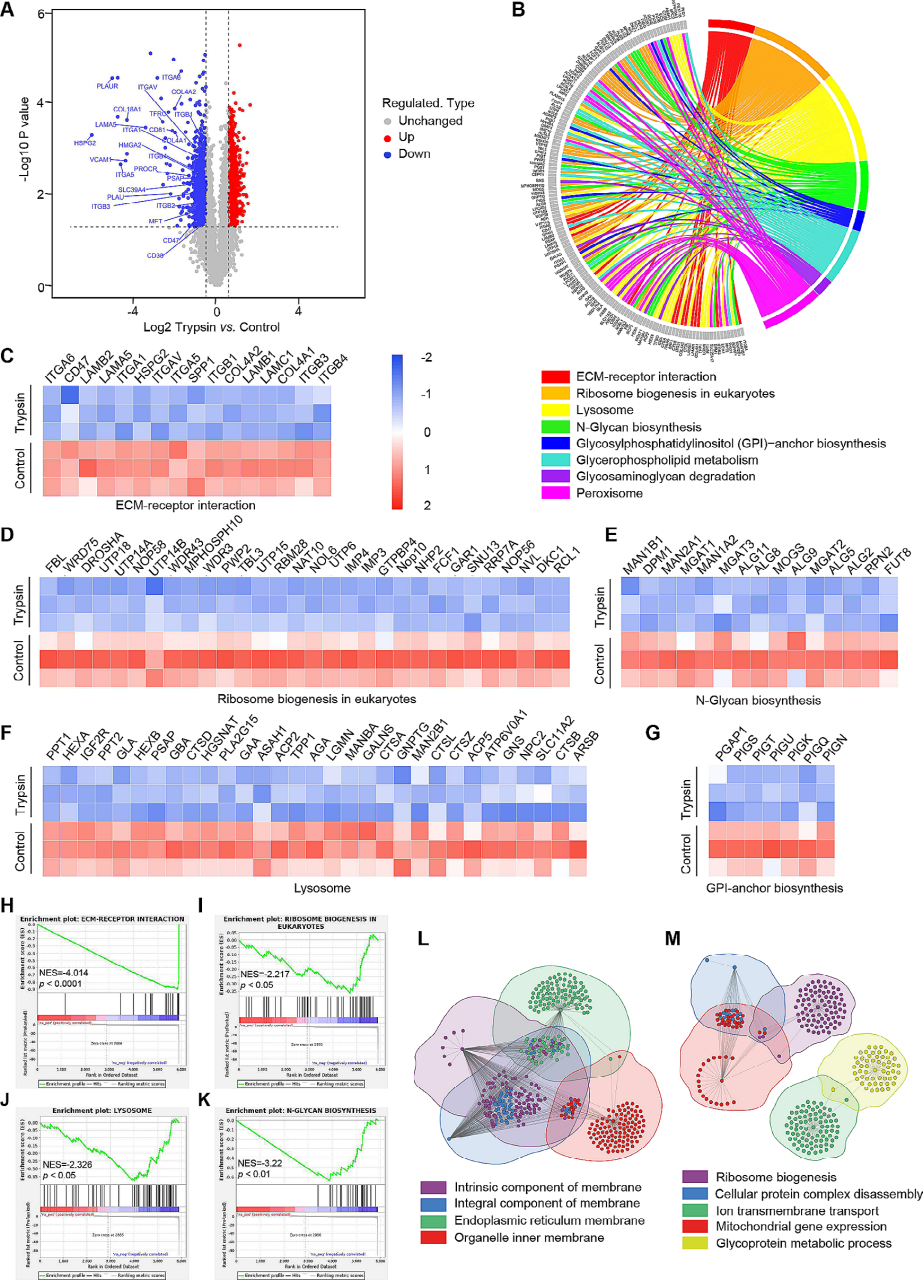
Proteomics profiling of 4T1 after 0.5% trypsin treatment for 30 min. (**A**) Volcano plots showing the down-regulated proteins before and after trypsin treatment. (**B**) Circos plot depicting major KEGG enrichment pathways. (**C**-**G**) Expression profiles in significant down-regulated KEGG enrichment pathways (ECM-receptor interaction, ribosome biogenesis in eukaryotes, N-Glycan biosynthesis, lysosome, and GPI-anchor biosynthesis) relative to proteolytic enzyme therapy. (**H**-**K**) GSEA enrichment analysis identifying the major KEGG pathways relative to proteolytic enzyme therapy. (**L**-**M**) Gene ontology enrichment analysis indicating the down-regulated proteins in (**L**) cellular component and (**M**) biological processes after trypsin enzymolysis

To gain insights into cellular network alterations in trypsin-treated 4T1 cells, we performed a label-free quantitative proteomic (PTM BIO, China) assessment (Fig. S11-S14). The results of significantly differentially expressed proteins in trypsin-treated cells compared with untreated controls are highlighted in the volcano plot shown in Fig. 4A. We identified over 6000 proteins in 4T1 cells with or without trypsin treatment (data not shown). As indicated previously, total and membrane proteins of 4T1 cells were degraded after 0.5% trypsin treatment for 30 min. To determine which pathway might be affected by trypsin, a pathway enrichment analysis of the identified 1207 down-regulated proteins was performed. Figure 4B-G and Fig. S15, identified typical significantly enriched pathways involved in the proteolytic enzyme therapy of tumor cells. For example, ECM-receptor interaction is highly enriched, where ECM composition proteins such as collagen, integrin, CD47, and laminin were significantly down-regulated. The composition of the ECM and cell-cell adhesion in solid tumors limit drug penetration and are associated with poor prognosis [43]. Trypsin treatment significantly reduced the protein levels of ECM, thus explaining the previously mentioned enhancements in distribution and penetration of nanomedicine in tumor. Other pathways related to tumor progressions, such as ribosome biogenesis and N-glycan biosynthesis, were also enriched. It was reported that ribosome biogenesis during cell cycle arrest fuels cellular plasticity and migration during EMT [44]. Targeting ribosome biogenesis might be a new strategy to treat aggressive metastatic cancers. Furthermore, N-glycosylation regulates the quality and magnitude of CAR T cell responses, paving the way for the rational design of improved therapies against solid malignancies [45]. Pathways involved in lysosome function were enriched as well. Specifically, V-ATPase and some of the lysosome acid hydrolases associated with its digestive function were down-regulated. The accumulation of undigested material causes the lysosome to swell, increasing the lysosomal membran’s permeability and eventually leading to cell death. Gene-set enrichment analysis (GSEA) implicates the down-regulation of those pathways as expected (Fig. 4H-K). As shown in Gene ontology (GO) enrichment analysis, the down-regulated proteins were associated with cellular components in cell membranes and organelle membranes, and biological processes such as GPI-anchor biosynthesis, ribosome biogenesis, cellular protein complex disassembly, ion transmembrane transport, mitochondrial gene expression, and glycoprotein metabolic process (Fig. 4L and M). Overall, trypsin treatment affected ECM components and subcellular organelles, therefore down-regulating multiple pathways in cancer progression, indicating the antitumor efficacy of proteolytic enzyme therapy.

### Construction and characterizations of Trypsin@PSA Gel

**Fig. 5:**
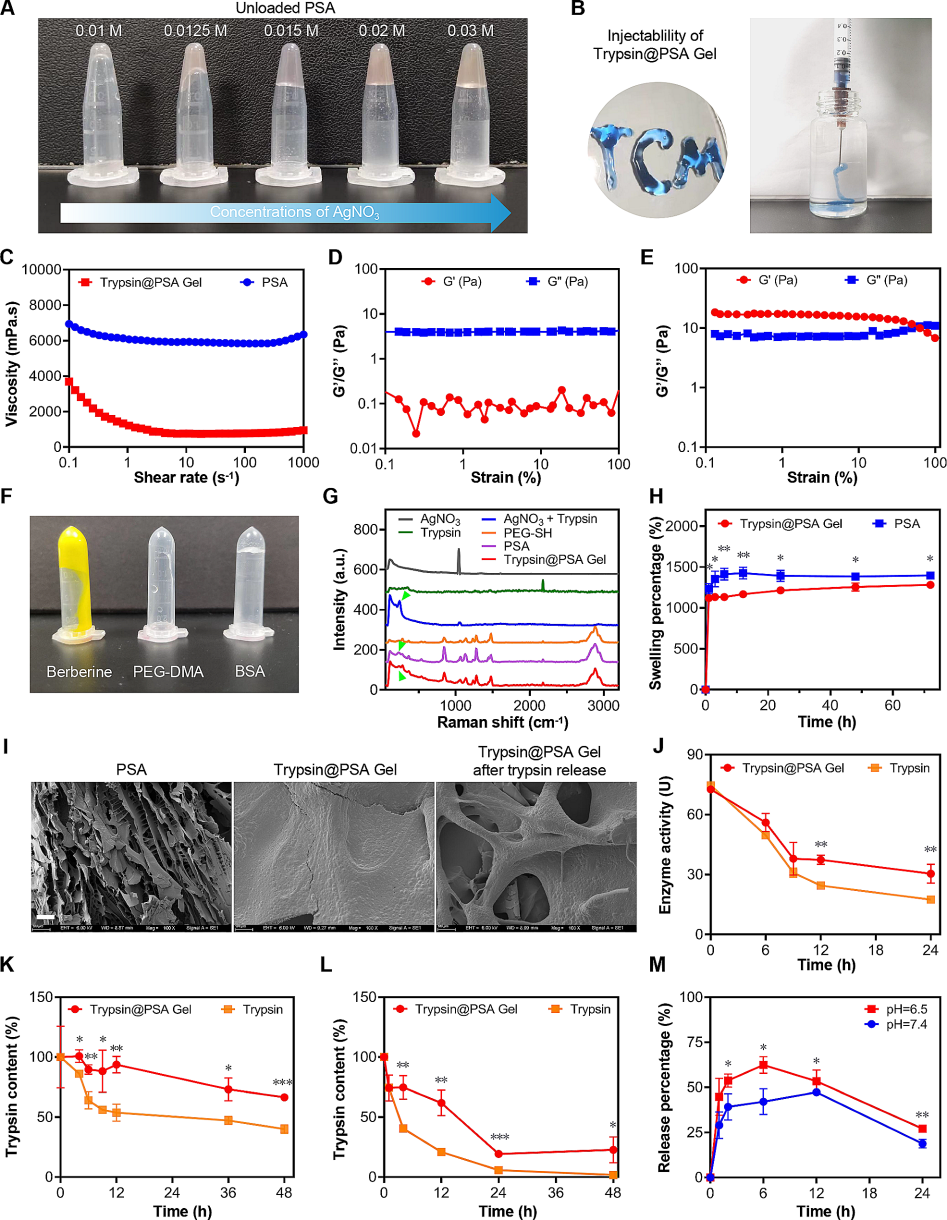
Construction and characterizations of Trypsin@PSA Gel. (**A**) The influence of AgNO_3_’s concentration on the gelation of unloaded PSA. (**B**) Injectability of Trypsin@PSA Gel loaded with 15 mg/mL trypsin. (**C**) The relationship between the viscosity of PSA or Trypsin@PSA Gel and shear rates. (**D**-**E**) Elastic moduli of (**D**) PSA and (**E**) Trypsin@PSA Gel (G′ and G″ represent storage modulus and loss modulus, respectively). (**F**) The influence of disulfide bond on PSA gel formation. (**G**) Raman spectra of AgNO_3_, trypsin, PEG-SH, the mixture of AgNO_3_ and trypsin, PSA, and Trypsin@PSA Gel (the green arrows indicate Ag-S bound). (**H**) The swelling percentage of PSA and Trypsin@PSA Gel. (**I**) Representative SEM images of PSA, Trypsin@PSA Gel loaded with 15 mg/mL trypsin, and Trypsin@PSA Gel after trypsin release, scale bar = 100 µm. (**J**) Trypsin activity in free form and Trypsin@PSA Gel at 37 ℃. (**K**-**L**) Trypsin stability in free form and Trypsin@PSA Gel at pH 6.5 (**K**) and 7.4 (**L**) at 37°C. (**M**) The release profile of trypsin from Trypsin@PSA Gel at different pH values at 37°C. All statistical data are presented as mean ± SD, *n* = 3. *p < 0.05, **p < 0.01, ***p < 0.001

We constructed a trypsin-loaded PEG hydrogel based on the commercially available PEG-SH with a star-like architecture and Ag^+^. Different concentrations of AgNO_3_ were incubated with trypsin and PEG-SH to explore the gelling condition (Fig. 5A). An optimal concentration of AgNO_3_ (0.015 mol/L) was selected to form the hydrogel. This concentration was chosen because it did not affect enzyme activity (Fig. S16). The injection capability of Trypsin@PSA Gel was tested. As shown in Fig. 5B, trypan blue-loaded hydrogel was continuously injected through the syringe with a 26 G needle, indicating the potential for in vivo tumor injection. Frequency sweep tests (Fig. 5C) were implemented between 0.1 and 1000 s^− 1^ to investigate the viscosity of the prepared hydrogel. It was observed that before applying a high shear rate, the hydrogel maintained its structure while keeping the viscosity at approximately 3600 mPa.s. In contrast, the viscosity of the hydrogel dropped to almost 800 mPa.s when a higher shear rate was applied, demonstrating the great injectability of the hydrogel. However, the viscosity of unloaded PSA (only containing PEG-SH and Ag^+^ without trypsin) did not change significantly. A Dynamic Mechanical Analysis (DMA) was performed to confirm the hydrogel formation. As shown in Fig. 5D and E, with the shear strain amplitude ranged from 0.1–100%, Trypsin@PSA Gel displayed the typical characteristics of gels with G′ (storage modulus) greater than G″ (loss modulus). Surprisingly, PEG-SH and 0.015 mol/L AgNO_3_ failed to form a hydrogel structure, with the loss modulus G″ lower than the storage modulus G′. This result indicates the contribution of trypsin on gel formation, which might be the Ag-S coordination with sulfhydryl groups in trypsin and Ag^+^.

To verify the hypothesis, PEG-SH and AgNO_3_ (0.015 mol/L) were incubated with a small molecule without disulfide bonds (berberine) and large molecules with or without disulfide bonds (bovine albumin (BSA) and poly (ethylene glycol) dimethacrylate (PEG-DMA), respectively). Pictures in Fig. 5F illustrate that only BSA formed a gel. Thus, it can be inferred that the disulfide bond is involved in the gelation process. Trypsin, by coincidence, is rich in cysteines-containing disulfide bonds (Fig. S1). Raman spectroscopy was applied to detect the characteristic peaks of the Ag-S bond in Trypsin@PSA Gel. Figure 5G shows the absorption peak of the Ag-S bond (green arrow) at 234 cm^− 1^ in Raman spectra of AgNO_3_ + Trypsin, PSA, and Trypsin@PSA Gel. However, they were not presented in other groups. This result suggests that the Ag-S bond was formed between PEG-SH or trypsin and Ag^+^ ; trypsin promoted the gelling process by forming disulfide bonds with Ag^+^. On the other hand, high-concentration AgNO_3_ facilitated gelation (Fig. 5A), whereas the involvement of trypsin reduced the concentration of AgNO_3_ used. Regarding the swelling percentages, Trypsin@PSA Gel exhibited a lower value than PSA. Specifically, Trypsin@PSA Gel reached swelling equilibrium at 72 h with a swelling ratio of 1200%. However, PSA reached swelling equilibrium at 12 h, and the swelling ratio is 1300% (Fig. 5H). The slow swelling process refers to a higher crosslink density; it suggests that the density of the internal network of Trypsin@PSA Gel is higher than PSA. The higher degree of crosslinking in Trypsin@PSA Gel with 5.3% trypsin loading (Fig. S17A) was also confirmed by SEM imaging. Trypsin@PSA Gel presented a dense structure, whereas porous structures were shown in PSA alone and Trypsin@PSA Gel after the release of trypsin (Fig. 5I).

Trypsin is a proteolytic enzyme with self-digesting capability in nature. Therefore, inhibiting the self-degradation of trypsin and maintaining the concentration of their action are necessary. Enzyme activity was detected at different time intervals at room temperature to test if the prepared hydrogel could protect trypsin from degradation. Figure 5J and Fig. S17B, reveal hydrogel’s protective effect on trypsin at 4 °C and 37 °C. As shown in Fig. 5K and L, compared with trypsin alone, Trypsin@PSA Gel significantly enhanced the stability of trypsin at pH 6.5 and 7.4. The choice of such pH values simulates a typical physiological environment and an extracellular acidic tumor microenvironment. Moreover, in both conditions, the trypsin release profile consisted of two stages: a sustained release followed by a slow degradation (Fig. 5M). Due to higher stability at a moderate acidic condition, more of the released trypsin was detected at pH 6.5. Although self-digestion is inevitable, the hydrogel maintained trypsin’s activities to some extent and postponed the self-degradation of trypsin in both conditions. Indeed, alive cells were detected after incubation with blank PSA but not Trypsin@PSA Gel (Fig. S18), indicating the good biocompatibility and the protein protective effect of hydrogel materials. These findings also reveal that trypsin is more stable at pH 6.5 and desirable for intratumoral injection.

### Trypsin@PSA Gel enhances nanoformulations penetration in solid tumor

**Fig. 6:**
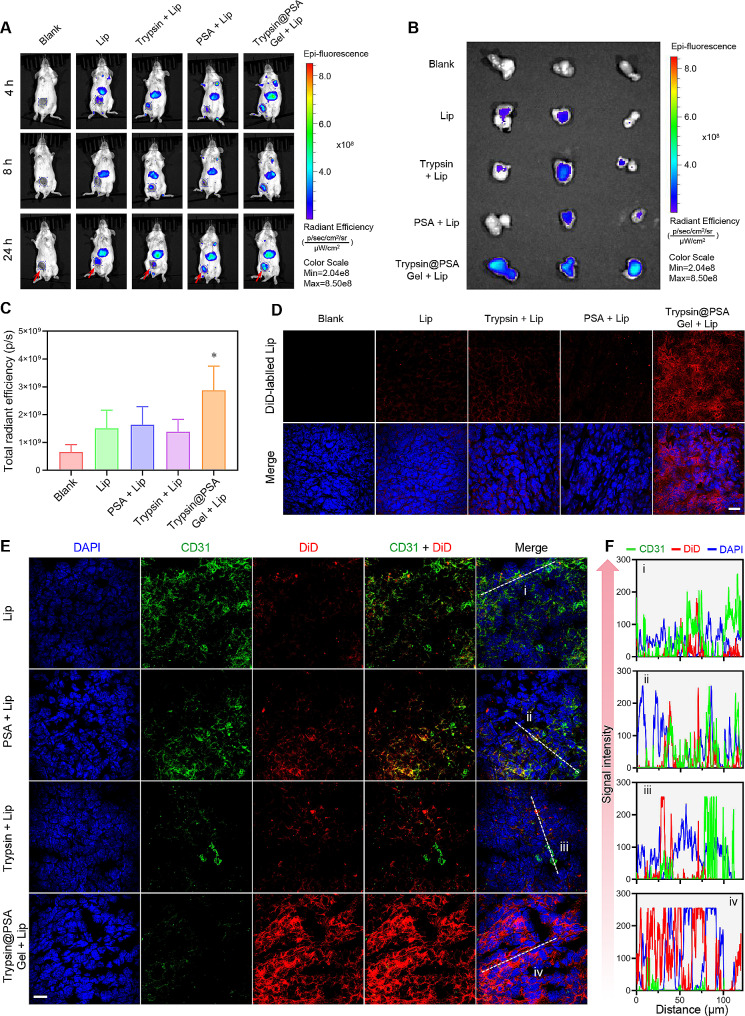
Proteolytic enzyme therapy facilitates the tumor distribution and penetration of liposomes. (**A**) Biodistribution of DiD-Lip in tumor-bearing mice with or without Trypsin@PSA Gel treatment (the red arrow indicates the implanted tumors). (**B**-**C**) Fluorescence images and total radiant efficiency of DiD in tumors at 48 h post-DiD-Lip injection. (**D**) Representative confocal images of tumor sections with or without Trypsin@PSA Gel treatment. (**E**) Representative confocal images showing CD31 expression and DiD-Lip location of tumor sections with or without Trypsin@PSA Gel treatment. (**F**) Quantification of the green (CD31) and red (DiD) fluorescence signals on the tumors as a function of distance (µm) marked by the white dotted lines in the confocal images in (**E**). All statistical data are presented as mean ± SD, *n* = 3. *p < 0.05, scale bar = 20 μm

To the best of our knowledge, the dilemma of most of the present anticancer drugs is the limited penetration in solid tumors [46, 47]. To achieve deep penetration in avascular tumor tissue, enhancing the permeability of tumor blood vessels and destroying tumor stroma are potential therapeutic strategies for nanomedicines. In our previous studies, trypsin contributed to the penetration of nanoformulations through the cell membrane and tight junctions by digesting membrane proteins of tumor cells. To verify the effect of trypsin on the deep penetration of nanoparticles, we performed a biodistribution study using DiD-labeled Lip (Fig. 6A). At 4 h, 8 h, and 24 h, DiD-Lip mainly accumulated in the liver, in concord with the consensus that most administered nanoparticles will accumulate and sequester in the liver after systemic administration. Interestingly, the accumulation of Lips in tumor tissues was notably visualized in the Trypsin@PSA Gel-treated group but was weakly observed in other groups (Fig. 6A-C and Fig. S19). The fluorescence signals of DiD were only increased slightly by trypsin treatment, probably owing to the partial deactivating of free trypsin in tumor tissues. However, injection of PSA alone failed to show any significant difference compared with the intravenous injection of DiD-Lip alone. Confocal laser scanning microscopy images of tumor sections in Fig. 6D also show an apparent distribution of DiD-Lip post-Trypsin@PSA Gel treatment. Interestingly, the proteolytic enzyme therapy treatment of solid tumors allowed DiD-Lip to penetrate deeper into the interior, whereas DiD-Lip gathered around tumors in groups without trypsin (Fig. S20). Further, we performed an immunofluorescence staining of tumor vessels with a CD31 antibody and analyzed the co-localization of the vessels and DiD-Lip (Fig. 6E). Intratumoral injection of trypsin alone and Trypsin@PSA Gel significantly reduced tumor vasculatures (Fig. 6E and F). Trypsin or PSA plus DiD-Lip did not contribute to the retention of DiD-labeled Lip within tumor parenchyma, with some of the Lip remaining in the blood vessels (yellow fluorescence). Notably, intratumoral injection of Trypsin@PSA Gel reduced blood vessels and significantly increased the retention of DiD-Lip in tumor parenchyma.

Following its discovery more than 30 years ago, the EPR effect has become the guiding principle for nanomedicine against cancer [47]. However, biological barriers, such as ECM, interstitial fluid pressure, and poor blood flow, may hinder the efficient implementation of the EPR effect for drug delivery. The dense ECM establishes a barrier inhibiting the movement of nanomedicine inside the tumor, severely resisting therapeutic agents’ penetration in the deep tumor tissues, resulting in poor efficacy and high tumor mortality. Thus, practical strategies to overcome the ECM barrier and enhance the nanoformulations penetration in solid tumors are highly required. Interestingly, trypsin erodes in vitro tumorsphere and in vivo parenchyma (Supplementary Video 1 and 2), like lava eroding a volcano, which may contribute to the improved nanoparticles penetration. Our discovery of promoting nanoformulations’ accumulation in solid tumors by proteolytic enzyme therapy may be a novel strategy for enhancing tumor targeting of nanomedicine.

### Trypsin@PSA Gel with GA-Lip prevents tumor growth

**Fig. 7:**
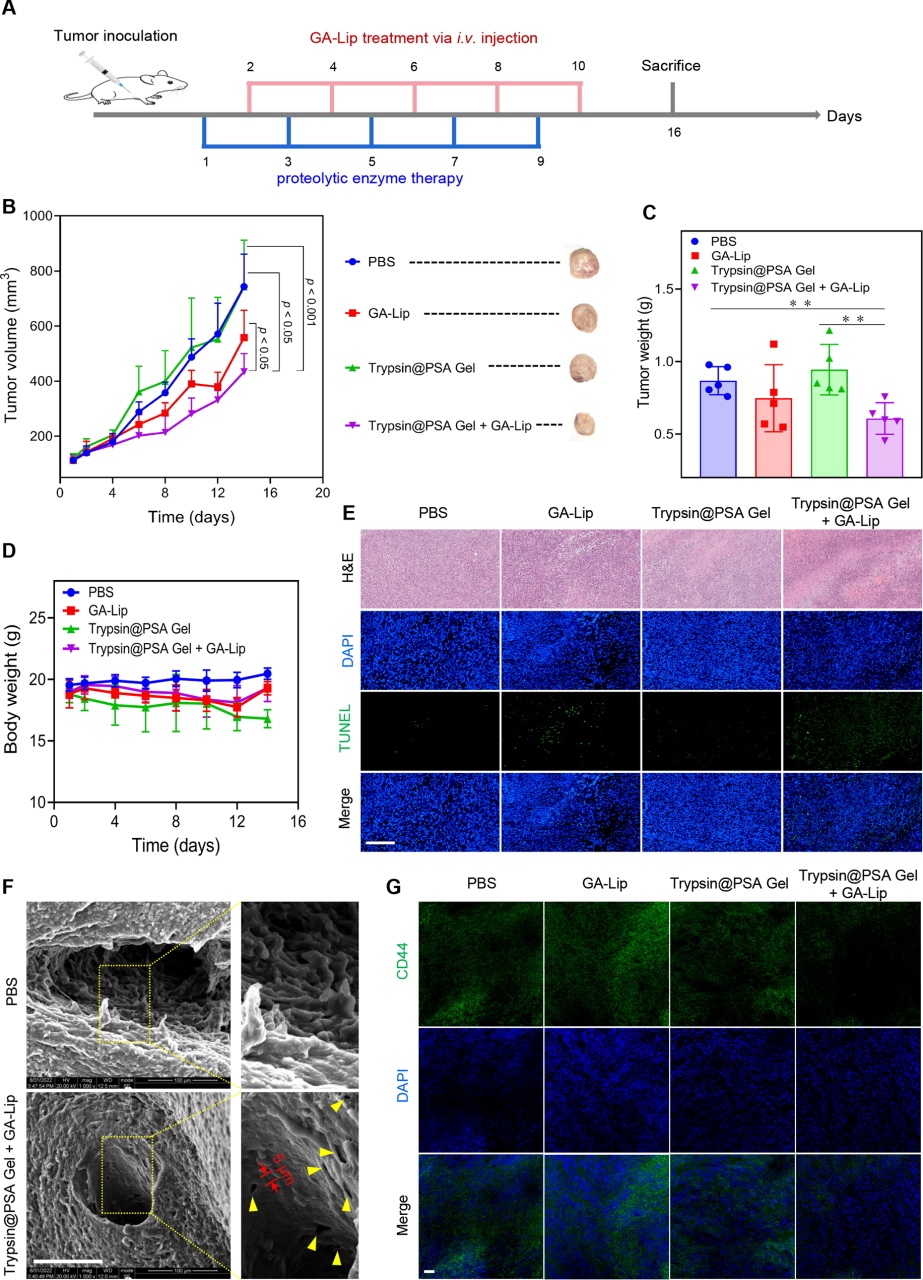
In vivo antitumor efficacy achieved by combination treatment with GA-Lip chemotherapy and proteolytic enzyme therapy. (**A**) Schematic illustration of the subcutaneous breast cancer model establishment and treatment. (**B**) Tumor volume variations during and after treatments (Insets indicate representative images of tumors after different treatments). (**C**) Weights of tumor after different treatments. (**D**) Body weights of mice during treatments. (**E**) H&E and TUNEL staining of tumor sections, scale bar = 250 μm. (**F**) Representative SEM images of tumor blood vessels after PBS or Trypsin@PSA + GA-Lip treatment, scale bar = 100 μm (the dashed square frames represent the lumen of the blood vessel, and the yellow arrows indicate the new-generated pores in blood vessels after proteolytic enzyme therapy). (**G**) Representative confocal images of CD44-stained tumor sections, scale bar = 50 μm. All statistical data are presented as mean ± SD, *n* = 5. *p < 0.05, **p < 0.01

A total of five injections of Trypsin@PSA Gel and GA-Lip were performed when the tumor reached 100–150 mm^3^ (Fig. 7A). As indicated in Fig. 7B, C and Fig. S21, combination treatment with Trypsin@PSA Gel and GA-Lip inhibited tumor growth compared with phosphate-buffered saline (PBS), GA-Lip, or Trypsin@PSA Gel treated groups. With the combination treatment, tumor-bearing mice achieved a much lower tumor weight than other groups. During the experiments, there are no significant differences between the weight of mice treated by GA-Lip + Trypsin@PSA Gel and PBS, indicating no significant toxicity (Fig. 7D).

In Fig. 7E, the tumor parenchyma by hematoxylin-eosin (H&E) staining became uneven in Trypsin@PSA Gel-injected groups, which was caused by the trypsin treatment. Additionally, the TUNEL assay reveals more apoptotic tumor cells in GA-Lip and the combination treatment groups. To investigate the effect of proteolytic enzyme therapy on tumor blood vessels, SEM images were captured to show the morphology of tumor blood vessels. As shown in Fig. 7F, in PBS-treated group, the tumor blood vessels were dilated with the irregular lumen. The vascular lumen, however, was much smoother after the combination treatment. This result suggests that treatment with trypsin may be beneficial to normalizing tumor blood vessels. In addition, some gaps, typically ∼ 5 μm in size, were present in the smooth vessels (Fig. 7F), which might also be evidence for trypsin’s digestion. Previously, we demonstrated the digestion of total and membrane proteins in tumor cells. Hence we suppose that some of the cell surface adhesion receptors promoting tumor progression, metastasis and chemoresistance were also degraded. CD44 was evaluated since it is highly expressed in cancers and plays a critical role in tumor progression and metastasis. In Fig. 7G and Fig. S22, cryo-sected tumor tissues post-treatments were stained with an anti-CD44 antibody (green). It is shown that the combination treatment remarkably decreased CD44 expression in 4T1 tumor tissue, whereas other treatments had little effect on its expression. Furthermore, the collagens in tumor stroma were also decreased after treatment with Trypsin@PSA Gel (Fig. S23). These findings indicate that digestive effect mediated by Trypsin@PSA Gel boosts the antitumor activity of nanomedicines. Healthy mice were injected with PBS and PSA subcutaneously for the biocompatibility study. Images of H&E-stained skin and muscle around the injection site showed no apparent inflammation (Fig. S24). As a result, it can be concluded that PSA is a safe material to load many other proteins containing sulfhydryl groups.

## Conclusion

Over the years, tumor-treated nanomedicine has made significant progress. However, the existence and the extent of the EPR effect, particularly in patients, are controversial. In this work, we conducted a trypsin-mediated proteolytic enzyme therapy to enhance nanoformulations’ penetration and retention. Trypsin accelerated the internalization of nanoformulations in mouse breast cancer 4T1 cell lines. In an in vitro tight junction model, trypsin contributed to the breakdown of endothelial tight junction and the penetration of fluorescence-labeled nanoformulations. Proteomic analysis reveals that trypsin influences multiple pathways in cancer progression. To minimize trypsin’s self-degradation and maintain its activities, we developed a hydrogel based on a trypsin-assisted crosslinking strategy. Trypsin contributed to gelation due to the formation of Ag-S bond with Ag^+^. The hydrogel protected trypsin from degradation and sustained its release at physiological and tumor microenvironmental pH. We observed an enhanced distribution and penetration of Lips in tumors pre-treated with Trypsin@PSA Gel through intratumoral injection. A phytochemical drug, GA, was loaded into Lip to evaluate the antitumor efficacy. The nanomedicine was injected intravenously after intratumoral injection with trypsin-loaded hydrogel. Finally, it was demonstrated that Trypsin@PSA Gel with GA-Lip chemotherapy prevented tumor growth. However, the current combination treatment’s therapeutic efficacy still needs improvement. This insufficiency may be remedied using a recombinant trypsin with more significant activities (≥ 4,500 U/mg) in place of the trypsin utilized in this study. Additionally, strategies to overcome the leakage of trypsin outside the tumor through intratumoral administration should also be considered. In summary, proteolytic enzyme therapy with trypsin-loaded hydrogel has the potential to overcome the limited EPR effect of nanomedicine. We believe that the emerging concept of proteolytic enzyme therapy has excellent prospects for treating other solid tumors.

### Electronic supplementary material

Below is the link to the electronic supplementary material.

Supplementary Material 1

## Data Availability

The data that support the findings of this study are available from the corresponding author upon reasonable request.

## Abbreviations

BSA: NP Bovine serum albumin nanoparticles
C6: Coumarin 6
DDS: Drug delivery systems
ECM: Extracellular matrix
EMT: Epithelial-to-mesenchymal
EPR: Enhanced permeability and retention effect
GA: Gambogic acid
GO: Gene ontology
GSEA: Gene-set enrichment analysis
KEGG: Kyoto encyclopedia of genes and genomes
Lip: Liposomes
PLGA NP: Poly (lactic-co-glycolic acid) nanoparticles
PSA Gel: Hydrogel containing PEG-SH and AgNO_3_

## References

[1] Zhao M, van Straten D, Broekman MLD, Préat V, Schiffelers RM (2020). Nanocarrier-based drug combination therapy for glioblastoma. Theranostics.

[2] Zhao M, Mi D, Ferdows BE, Li Y, Wang R, Li J, Patel D, Kong N, Shi S, Tao W (2022). State-of-the-art nanotechnologies for the detection, recovery, analysis and elimination of liquid biopsy components in cancer. Nano Today.

[3] Zhao M, Wang R, Yang K, Jiang Y, Peng Y, Li Y, Zhang Z, Ding J, Shi S (2023). Nucleic acid nanoassembly-enhanced RNA therapeutics and diagnosis. Acta Pharm Sin B.

[4] Wong KH, Yang D, Chen S, He C, Chen M (2022). Development of nanoscale drug delivery systems of dihydroartemisinin for cancer therapy: a review. Asian J Pharm Sci.

[5] Dong S, Ma S, Chen H, Tang Z, Song W, Deng M (2022). Nucleobase-crosslinked poly(2-oxazoline) nanoparticles as paclitaxel carriers with enhanced stability and ultra-high drug loading capacity for breast cancer therapy. Asian J Pharm Sci.

[6] Matsumura Y, Maeda H (1986). A new concept for macromolecular therapeutics in cancer chemotherapy: mechanism of tumoritropic accumulation of proteins and the antitumor agent smancs. Cancer Res.

[7] Shi Y, van der Meel R, Chen X, Lammers T (2020). The EPR effect and beyond: strategies to improve tumor targeting and cancer nanomedicine treatment efficacy. Theranostics.

[8] Fang J, Islam W, Maeda H (2020). Exploiting the dynamics of the EPR effect and strategies to improve the therapeutic effects of nanomedicines by using EPR effect enhancers. Adv Drug Deliv Rev.

[9] Ikeda-Imafuku M, Wang LL, Rodrigues D, Shaha S, Zhao Z, Mitragotri S (2022). Strategies to improve the EPR effect: a mechanistic perspective and clinical translation. J Control Release.

[10] Matsumura Y (2020). Cancer stromal targeting therapy to overcome the pitfall of EPR effect. Adv Drug Deliv Rev.

[11] Matsumura Y, Gotoh M, Muro K, Yamada Y, Shirao K, Shimada Y, Okuwa M, Matsumoto S, Miyata Y, Ohkura H (2004). Phase I and pharmacokinetic study of MCC-465, a doxorubicin (DXR) encapsulated in PEG immunoliposome, in patients with metastatic stomach cancer. Ann Oncol.

[12] Zhang Y, Liu Y, Gao X, Li X, Niu X, Yuan Z, Wang W (2019). Near-infrared-light induced nanoparticles with enhanced tumor tissue penetration and intelligent drug release. Acta Biomater.

[13] Wang X, Zhang H, Chen X, Wu C, Ding K, Sun G, Luo Y, Xiang D (2023). Overcoming tumor microenvironment obstacles: current approaches for boosting nanodrug delivery. Acta Biomater.

[14] Zinger A, Koren L, Adir O, Poley M, Alyan M, Yaari Z, Noor N, Krinsky N, Simon A, Gibori H (2019). Collagenase nanoparticles enhance the penetration of drugs into pancreatic tumors. ACS Nano.

[15] Huang HY, Chen LQ, Sun W, Du HH, Dong S, Ahmed AMQ, Cao D, Cui JH, Zhang Y, Cao QR (2021). Collagenase IV and clusterin-modified polycaprolactone-polyethylene glycol nanoparticles for penetrating dense tumor tissues. Theranostics.

[16] Carver K, Ming X, Juliano RL (2014). Tumor cell-targeted delivery of nanoconjugated oligonucleotides in composite spheroids. Nucleic Acid Ther.

[17] Sindhwani S, Syed AM, Ngai J, Kingston BR, Maiorino L, Rothschild J, MacMillan P, Zhang Y, Rajesh NU, Hoang T (2020). The entry of nanoparticles into solid tumours. Nat Mater.

[18] González-Titos A, Hernández-Camarero P, Barungi S, Marchal JA, Kenyon J, Perán M (2021). Trypsinogen and chymotrypsinogen: potent anti-tumor agents. Expert Opin Biol Ther.

[19] Isaacs LL (2022). Pancreatic proteolytic enzymes and cancer: new support for an old theory. Integr Cancer Ther.

[20] Gremmler L, Kutschan S, Dörfler J, Büntzel J, Büntzel J, Hübner J (2021). Proteolytic enzyme therapy in complementary oncology: a systematic review. Anticancer Res.

[21] Leipner J, Saller R (2000). Systemic enzyme therapy in oncology: effect and mode of action. Drugs.

[22] Yamamoto K, Morikawa K, Imanaka H, Imamura K, Kitamori T (2020). Picoliter enzyme reactor on a nanofluidic device exceeding the bulk reaction rate. Analyst.

[23] Qin X, Yu C, Wei J, Li L, Zhang C, Wu Q, Liu J, Yao SQ, Huang W (2019). Rational design of nanocarriers for intracellular protein delivery. Adv Mater.

[24] Liu J, Ding X, Fu Y, Xiang C, Yuan Y, Zhang Y, Yu P (2021). Cyclodextrins based delivery systems for macro biomolecules. Eur J Med Chem.

[25] Li H, Gou R, Liao J, Wang Y, Qu R, Tang Q, Gan J, Zou L, Shi S (2023). Recent advances in nano-targeting drug delivery systems for rheumatoid arthritis treatment. Acta Materia Med.

[26] van de Weert M, Hennink WE, Jiskoot W (2000). Protein instability in poly(lactic-co-glycolic acid) microparticles. Pharm Res.

[27] Ye T, Wang J, Jiao Y, Li L, He E, Wang L, Li Y, Yun Y, Li D, Lu J (2022). A tissue-like soft all-hydrogel battery. Adv Mater.

[28] Chen W, Tao W (2022). Precise control of the structure of synthetic hydrogel networks for precision medicine applications. Matter.

[29] Feng C, Ouyang J, Tang Z, Kong N, Liu Y, Fu L, Ji X, Xie T, Farokhzad OC, Tao W (2020). Germanene-based theranostic materials for surgical adjuvant treatment: inhibiting tumor recurrence and wound infection. Matter.

[30] Shen C, Jiang T, Lou Q, Zhao W, Lv C, Zheng G, Liu H, Li P, Dai L, Liu K (2022). Near-infrared chemiluminescent carbon nanogels for oncology imaging and therapy. SmartMat.

[31] Mi D, Li J, Wang R, Li Y, Zou L, Sun C, Yan S, Yang H, Zhao M, Shi S (2023). Postsurgical wound management and prevention of triple-negative breast cancer recurrence with a pryoptosis-inducing, photopolymerizable hydrogel. J Control Release.

[32] Wang X, Wang Q (2021). Enzyme-laden bioactive hydrogel for biocatalytic monitoring and regulation. Acc Chem Res.

[33] Na K, Liu K, Yu J, Wang X, Li M, Tian C, He H, He Y, Wang Y (2020). A solvent-assisted active loading technology to prepare gambogic acid and all-trans retinoic acid co-encapsulated liposomes for synergistic anticancer therapy. Drug Deliv Transl Res.

[34] Li J, Wei J, Wan Y, Du X, Bai X, Li C, Lin Y, Liu Z, Zhou M, Zhong Z (2021). TAT-modified tetramethylpyrazine-loaded nanoparticles for targeted treatment of spinal cord injury. J Control Release.

[35] Zhao M, Bozzato E, Joudiou N, Ghiassinejad S, Danhier F, Gallez B, Préat V (2019). Codelivery of paclitaxel and temozolomide through a photopolymerizable hydrogel prevents glioblastoma recurrence after surgical resection. J Control Release.

[36] Wang X, Yu JY, Sun Y, Wang H, Shan H, Wang S (2021). Baicalin protects LPS-induced blood-brain barrier damage and activates Nrf2-mediated antioxidant stress pathway. Int Immunopharmacol.

[37] Gaillard PJ, Voorwinden LH, Nielsen JL, Ivanov A, Atsumi R, Engman H, Ringbom C, de Boer AG, Breimer DD (2001). Establishment and functional characterization of an in vitro model of the blood-brain barrier, comprising a co-culture of brain capillary endothelial cells and astrocytes. Eur J Pharm Sci.

[38] Shen Z, Ye H, Kröger M, Li Y (2018). Aggregation of polyethylene glycol polymers suppresses receptor-mediated endocytosis of PEGylated liposomes. Nanoscale.

[39] Malinovskaya Y, Melnikov P, Baklaushev V, Gabashvili A, Osipova N, Mantrov S, Ermolenko Y, Maksimenko O, Gorshkova M, Balabanyan V (2017). Delivery of doxorubicin-loaded PLGA nanoparticles into U87 human glioblastoma cells. Int J Pharm.

[40] Zhang X, Xu Y, Zhang W, Fu X, Hao Z, He M, Trefilov D, Ning X, Ge H, Chen Y (2020). Controllable subtractive nanoimprint lithography for precisely fabricating paclitaxel-loaded PLGA nanocylinders to enhance anticancer efficacy. ACS Appl Mater Interfaces.

[41] Engin AB, Nikitovic D, Neagu M, Henrich-Noack P, Docea AO, Shtilman MI, Golokhvast K, Tsatsakis AM (2017). Mechanistic understanding of nanoparticles’ interactions with extracellular matrix: the cell and immune system. Part Fibre Toxicol.

[42] Stoller JK, Aboussouan LS (2005). Alpha1-antitrypsin deficiency. Lancet.

[43] Minchinton AI, Tannock IF (2006). Drug penetration in solid tumours. Nat Rev Cancer.

[44] Prakash V, Carson BB, Feenstra JM, Dass RA, Sekyrova P, Hoshino A, Petersen J, Guo Y, Parks MM, Kurylo CM (2019). Ribosome biogenesis during cell cycle arrest fuels EMT in development and disease. Nat Commun.

[45] Greco B, Malacarne V, De Girardi F, Scotti GM, Manfredi F, Angelino E, Sirini C, Camisa B, Falcone L, Moresco MA (2022). Disrupting N-glycan expression on tumor cells boosts chimeric antigen receptor T cell efficacy against solid malignancies. Sci Transl Med.

[46] Sun R, Xiang J, Zhou Q, Piao Y, Tang J, Shao S, Zhou Z, Bae YH, Shen Y (2022). The tumor EPR effect for cancer drug delivery: current status, limitations, and alternatives. Adv Drug Deliv Rev.

[47] Zhou Q, Shao S, Wang J, Xu C, Xiang J, Piao Y, Zhou Z, Yu Q, Tang J, Liu X (2019). Enzyme-activatable polymer-drug conjugate augments tumour penetration and treatment efficacy. Nat Nanotechnol.

