# Supplementary material for: A lava-inspired proteolytic enzyme therapy on cancer with a PEG-based hydrogel enhances tumor distribution and penetration of liposomes
Source: J Nanobiotechnology. 2024 May 2;22:216. doi: 10.1186/s12951-024-02468-7 (PMC11067103; doi:10.1186/s12951-024-02468-7)
Supplement: Supplementary file 1 — Supplementary Material 1 [file 12951_2024_2468_MOESM1_ESM.docx]

Supplemental information

A lava-inspired proteolytic enzyme therapy on cancer with a PEG-based hydrogel enhances tumor distribution and penetration of liposomes

*Jiaojiao Li†, Dandan Mi†, Rujing Wang, Yuke Li, Mengnan Zhao* and Sanjun Shi**

*State Key Laboratory of Southwestern Chinese Medicine Resources, School of Pharmacy, Chengdu University of Traditional Chinese Medicine, China*

† Jiaojiao Li and Dandan Mi contributed equally to this work.

* Correspondence:

Mengnan Zhao: zhaomengnan@cdutcm.edu.cn

Sanjun Shi: shisanjuns@cdutcm.edu.cn and shisanjuns@163.com

State Key Laboratory of Southwestern Chinese Medicine Resources, School of Pharmacy, Chengdu University of Traditional Chinese Medicine, Chengdu 611137, China.

**Supplemental results:**


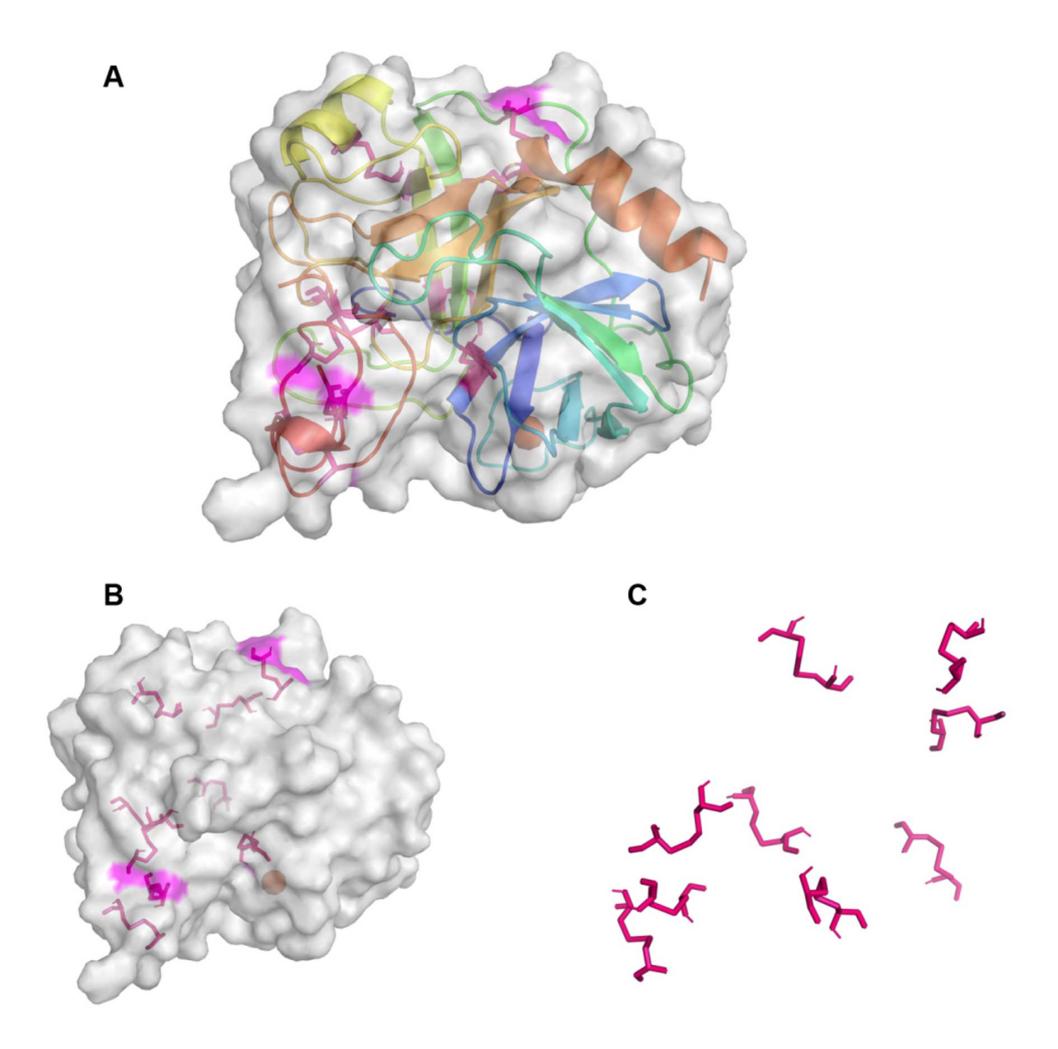


**Fig. S1** (A) Structure of porcine trypsin in complex with MCTI-A (a trypsin inhibitor of squash family) from Protein Data Bank with a PDB code of 1MCT ^[1]^. (B-C) The cysteines (-SH) are found in trypsin protein. Rainbow ribbons and white surface: trypsin and inhibitor complex. Pink sticks and surface: cysteine. Orange ball: calcium ion.

**Table S1** Physical characterazation of GA nanoformulations (*n* = 3, mean ± SD).

|  | Size (nm) | PDI | Zeta potential (mV) |
| --- | --- | --- | --- |
| GA-Lip | 136.59 ± 1.72 | 0.182 ± 0.017 | -19.37 ± 0.55 |
| GA-BSA NP | 113.79 ± 5.60 | 0.136 ± 0.016 | -2.01 ± 0.81 |
| GA-PLGA NP | 209.15 ± 11.59 | 0.053 ± 0.039 | -2.57 ± 0.29 |

**
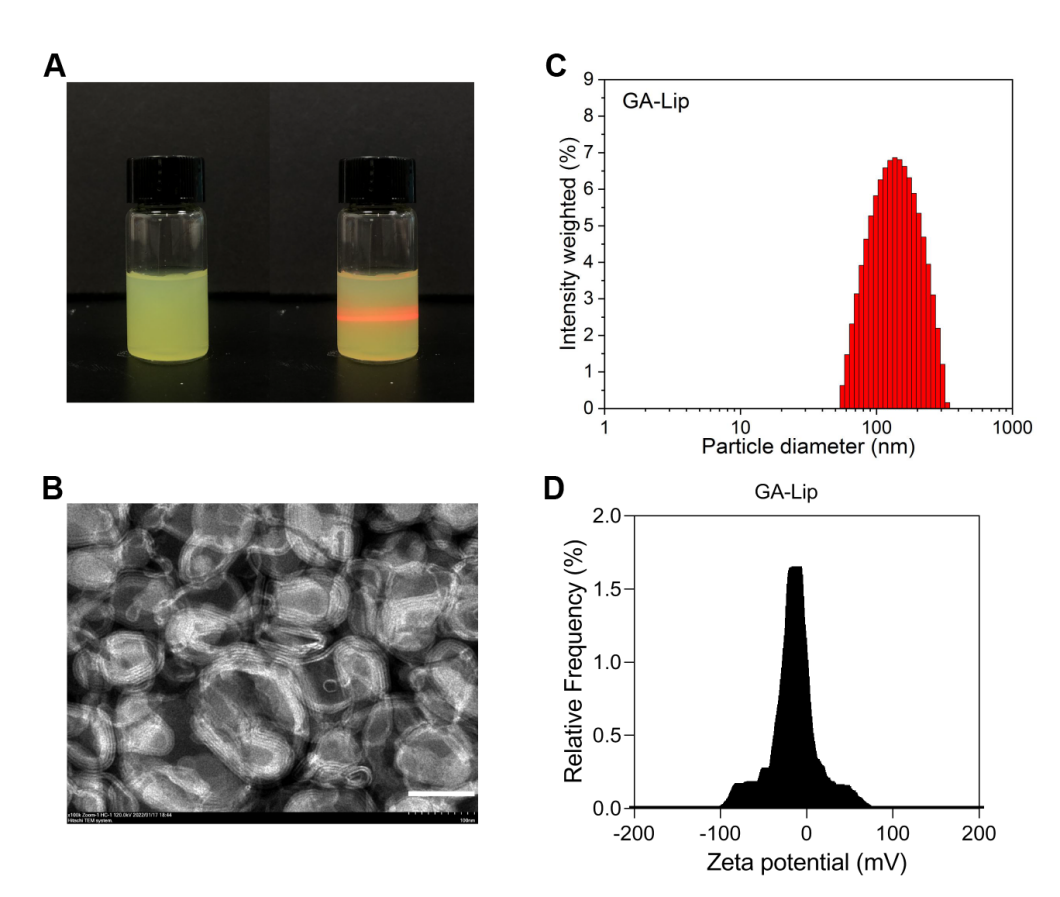
**

**Fig. S2** Charecterizations of GA loaded liposomes (GA-Lip). (A) Representative appearance of GA-Lip and its tyndall effect. (B) Representative TEM images showing the morphology of GA-Lip. Scale bar = 100 nm. (C) Particle diameter of GA-Lip conducted by DLS measurement. (D) Zeta potential of GA-Lip.


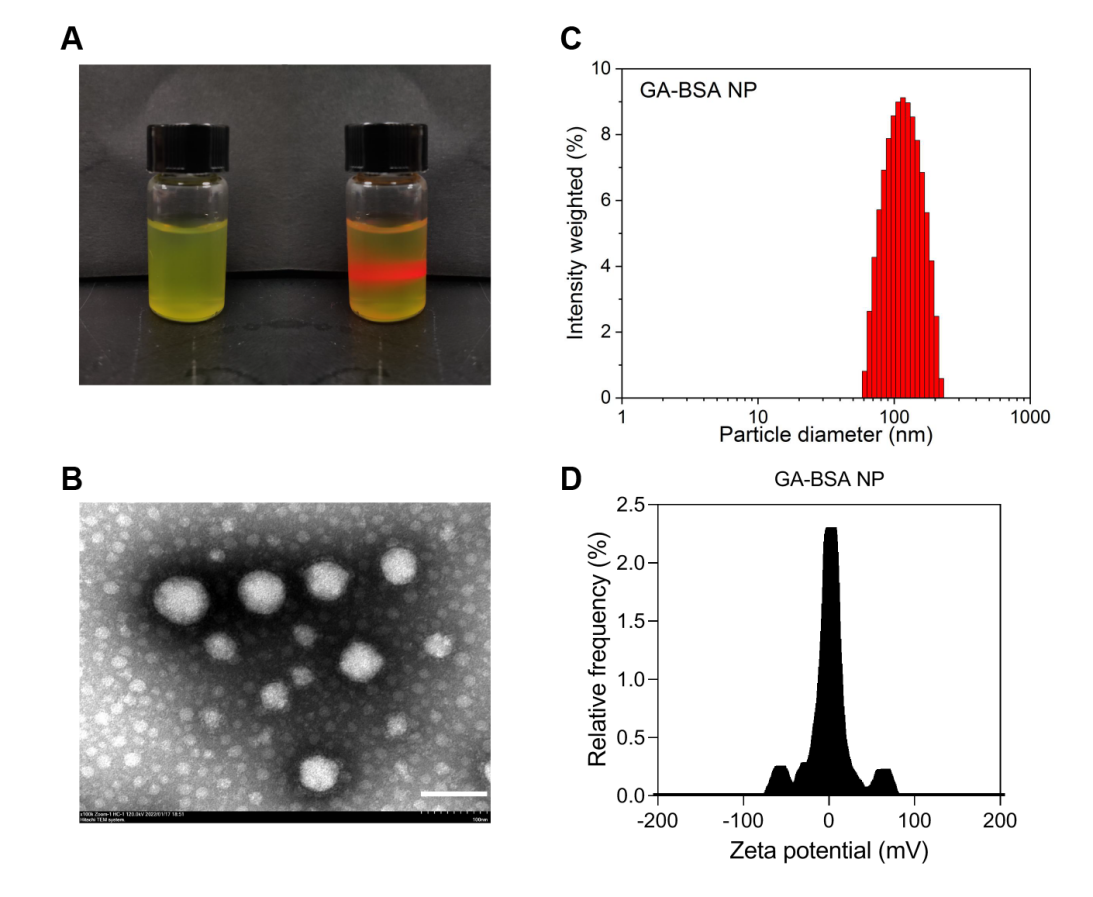


**Fig. S3** Charecterizations of GA loaded BSA nanoparticles (GA-BSA NP). (A) Representative appearance of GA-BSA NP and its tyndall effect. (B) Representative TEM images showing the morphology of GA-BSA NP. Scale bar = 100 nm. (C) Particle diameter of GA-BSA NP by DLS measurement. (D) Zeta potential of GA-BSA NP.

**
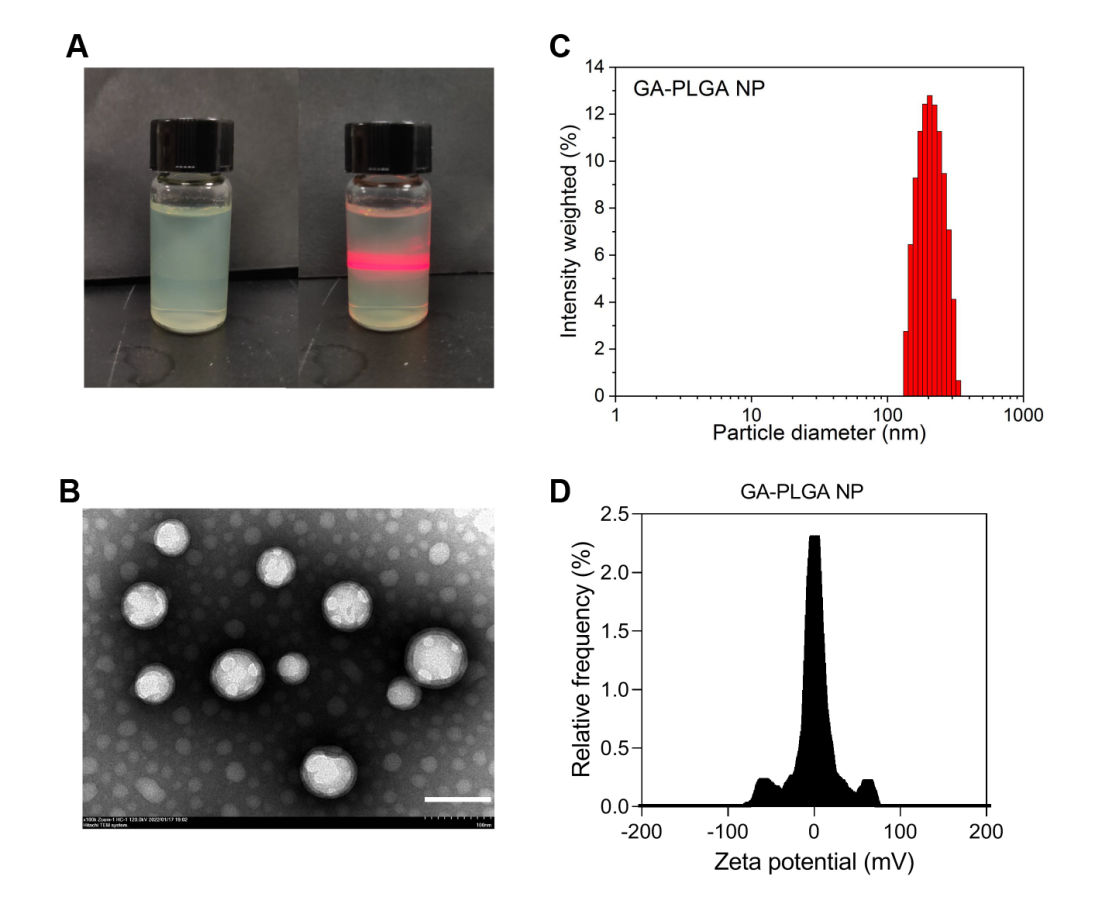
**

**Fig. S4** Charecterizations of GA loaded PLGA nanoparticles (GA-PLGA NP). (A) Representative appearance of GA-PLGA NP and its tyndall effect. (B) Representative TEM images showing the morphology of GA-PLGA NP. Scale bar = 100 nm. (C) Particle diameter of GA-PLGA NP by DLS measurement. (D) Zeta potential of GA-PLGA NP.


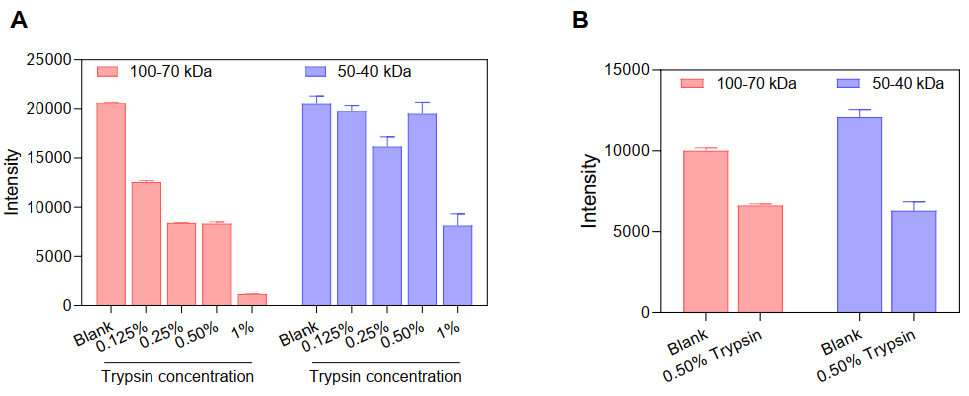


**Fig. S5** Quantitative results in Fig. 2B (A) and C (B).

***
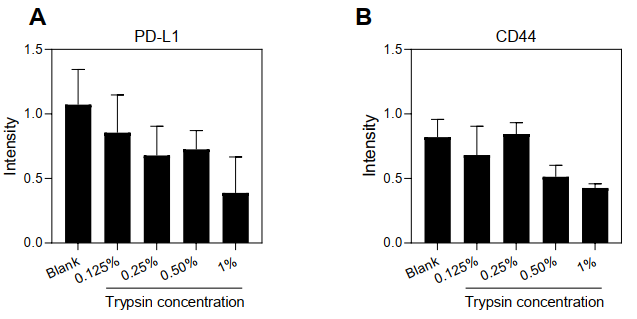
***

**Fig. S6** Quantitative results in Fig. 2E (A) and F (B).


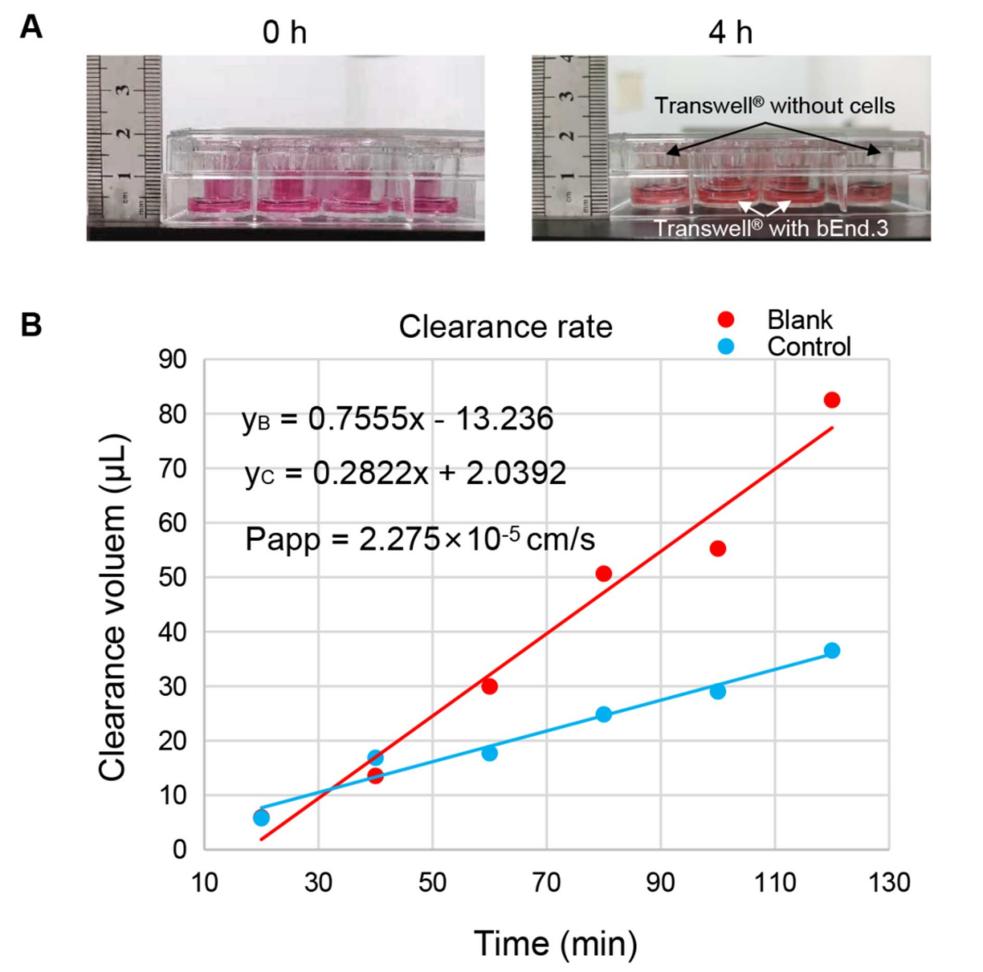


**Fig. S7** Confirmation of bEnd.3 tight junction formation. (A) Results of 4 h leakage test. (B) Results of sodium fluorescein permeability.


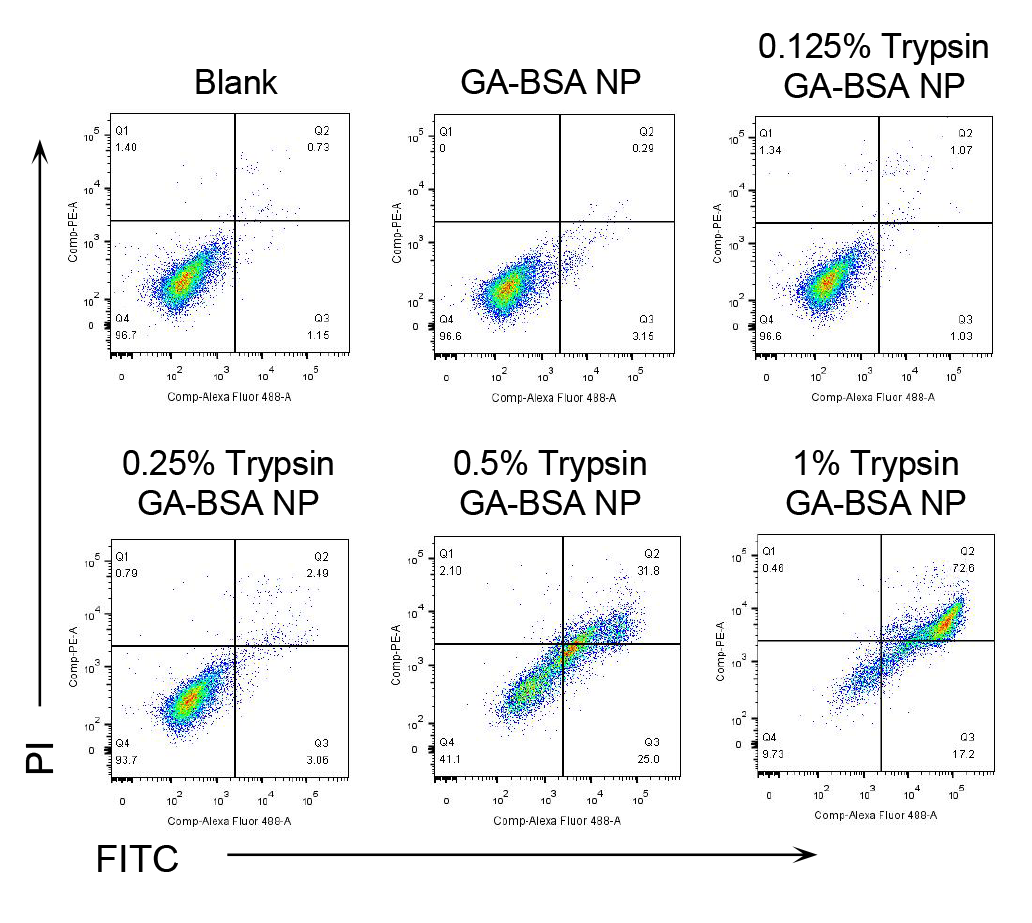


**Fig. S8** Flow cytometric analysis of cell apoptosis with GA-BSA NP administration after trypsin pretreatments. The experiments were conducted three times independently.


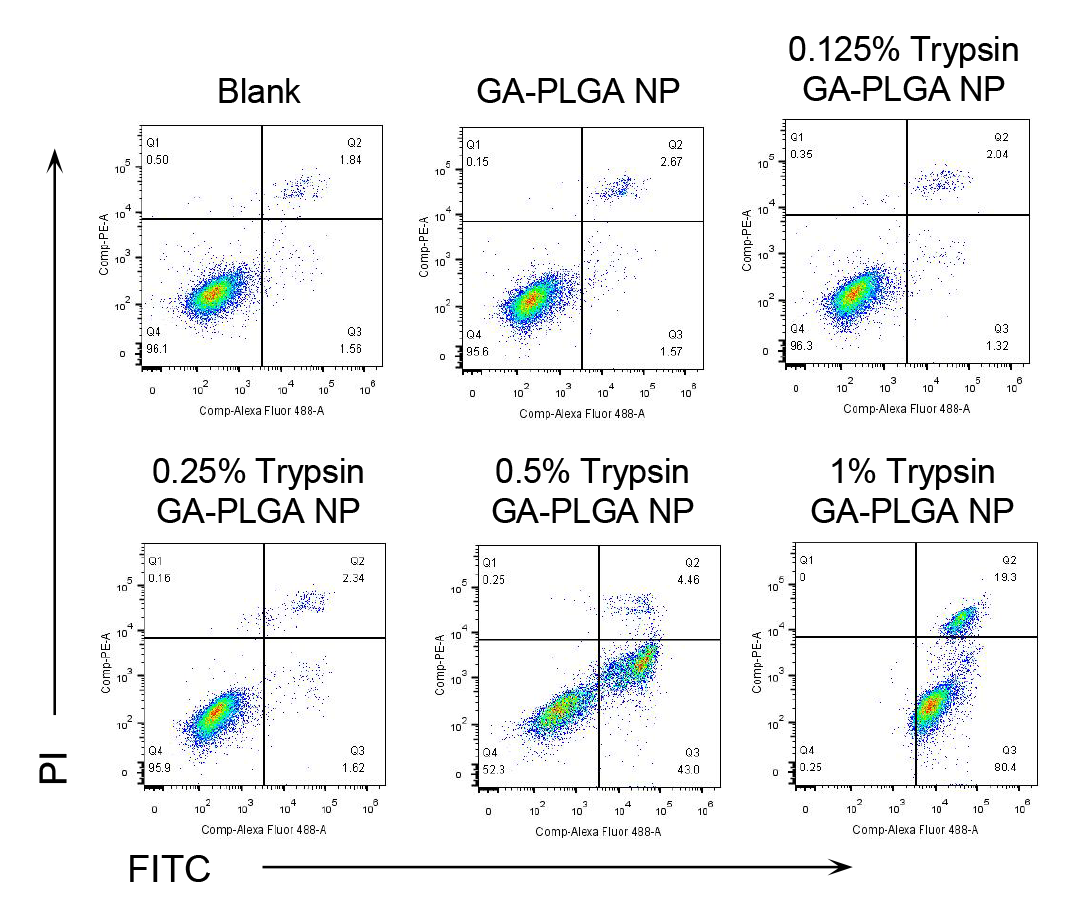


**Fig. S9** Flow cytometric analysis of cell apoptosis with GA-PLGA NP administration after trypsin pretreatments. The experiments were conducted three times independently.


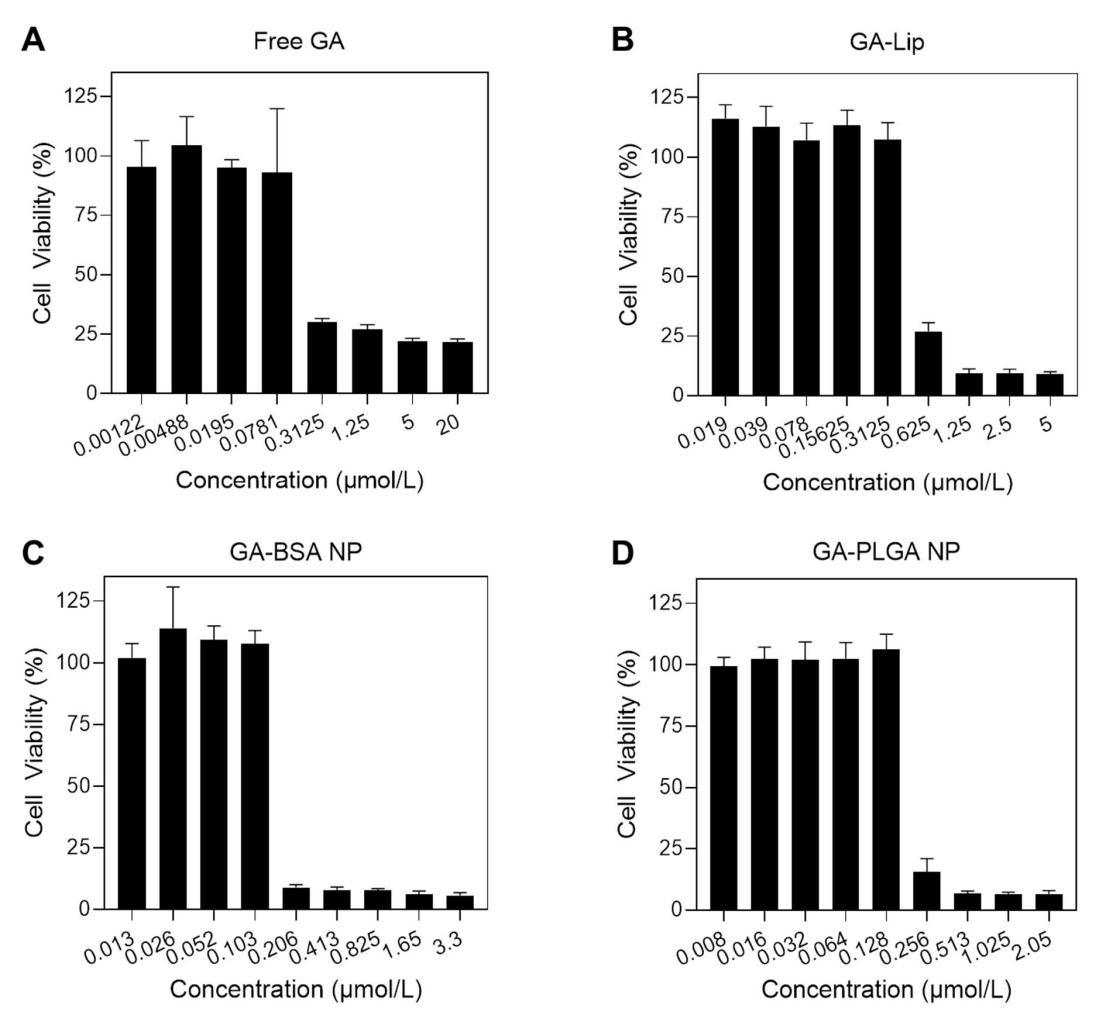


**Fig. S10** Cell viability of 4T1 after treatments with (A) GA or GA nanoformulations including: (B) GA-Lip, (C) GA-BSA NP, and (D) GA-PLGA NP. The experiments were conducted three times independently. All data are presented as mean ± SD, *n* = 4.

**
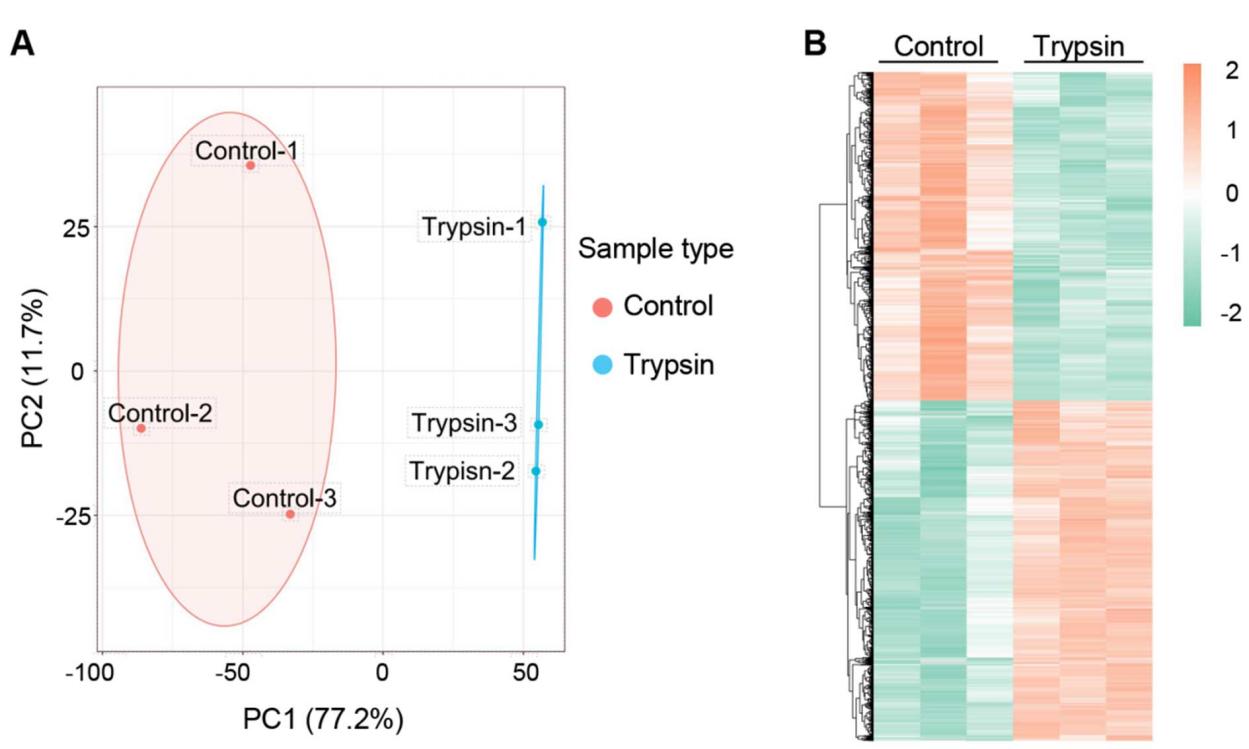
**

**Fig. S11** Proteomics analysis of 4T1 cells before and after 0.5% trypsin digestion for 30 min. (A) Principal component analysis (PCA) plots indicate that the repeated samples are statistically consistent. (B) Heat map of significantly different proteins. There was a total of 2457 altered proteins, with 1207 being down-regulated and 1250 being up-regulated. The experiments were conducted three times independently.

**
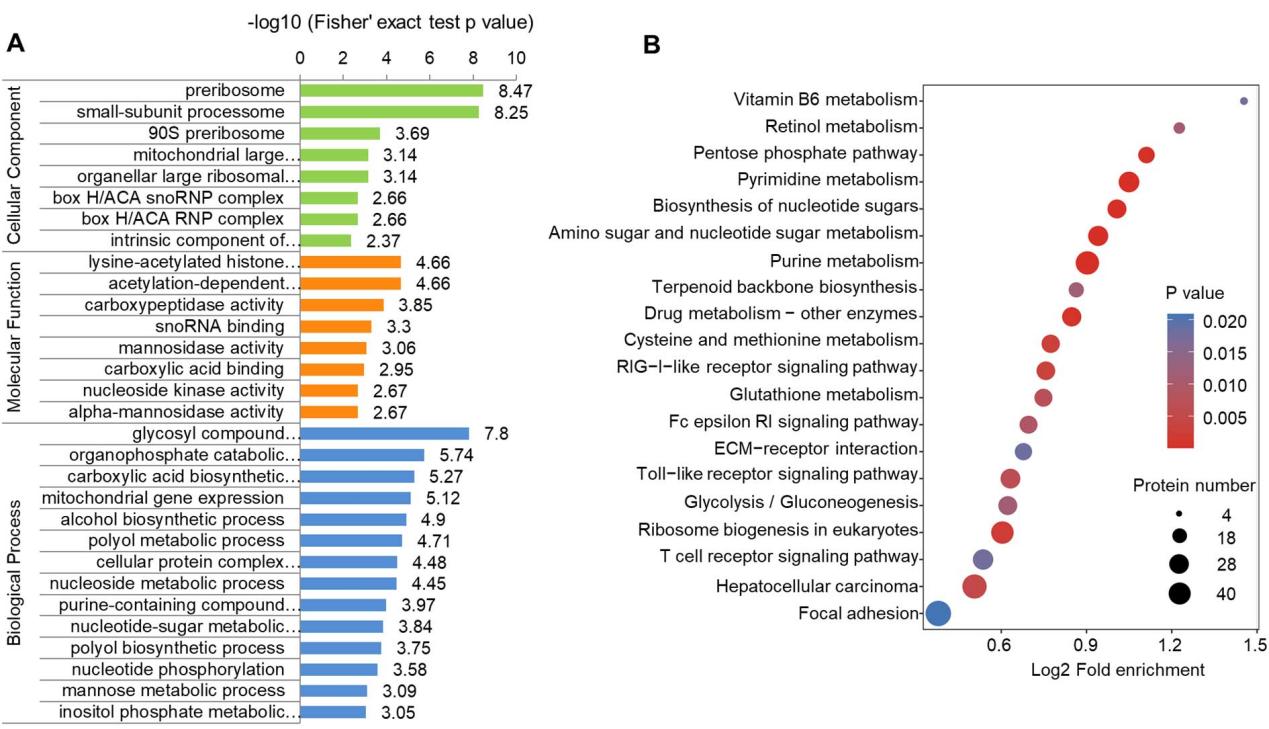
**

**Fig. S12** (A) GO enrichment analysis indicates all the altered proteins in cellular components, molecular function, and biological processes after trypsin digestion. (B) KEGG pathway enrichment analysis showing the up-regulated or down-regulated pathways after being treated with trypsin.


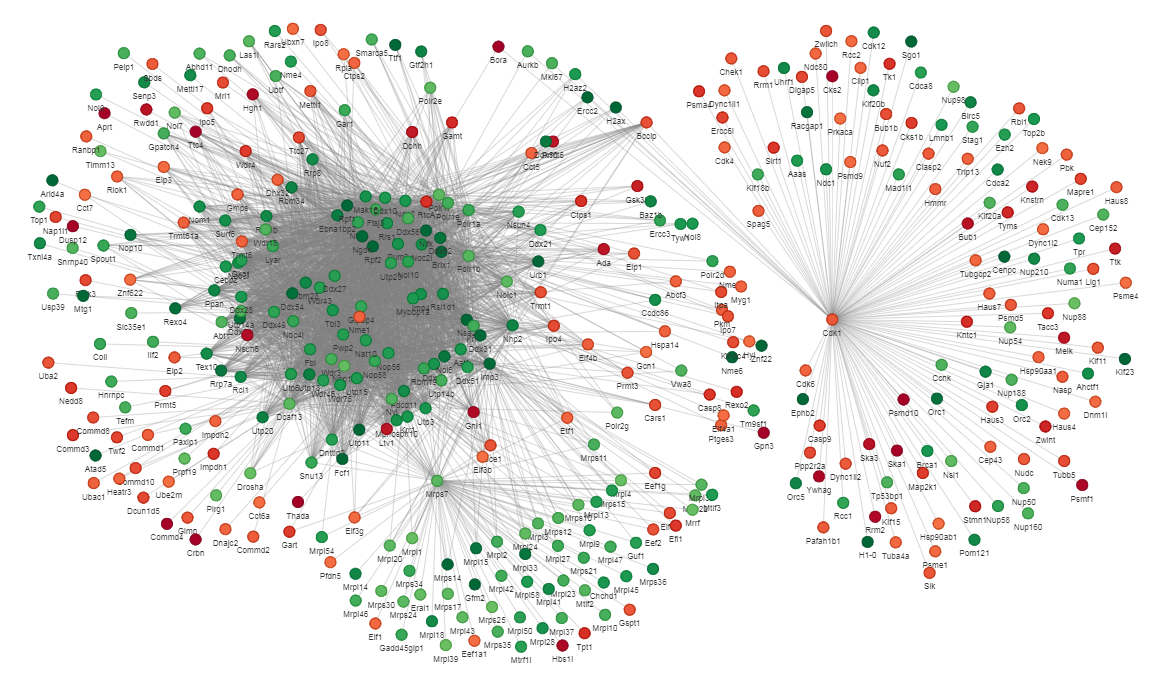


**Fig. S13** PPI network of proteins after trypsin digestion.


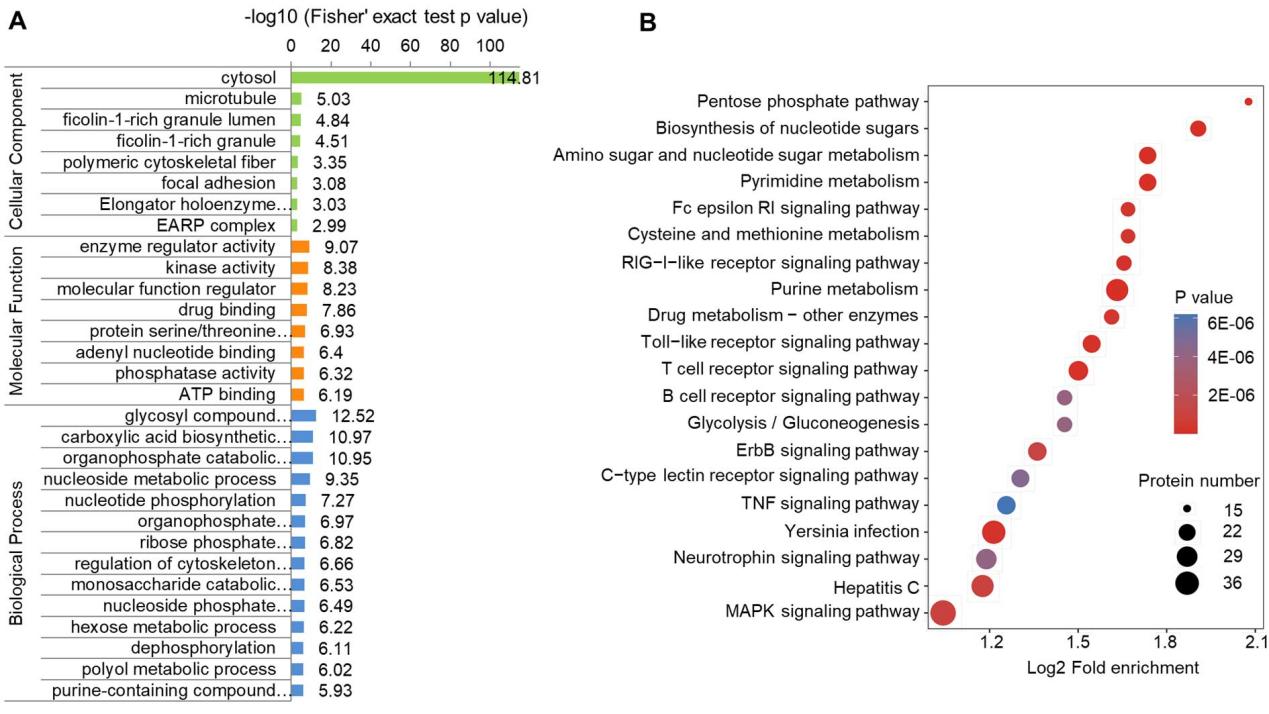


**Fig. S14** Proteomics analysis showing up-regulated proteins in 4T1 cells after 0.5% trypsin treated for 30 min. (A) GO enrichment analysis indicates the up-regulated proteins in cellular components, molecular function and biological processes. (B) KEGG pathway enrichment analysis showing the up-regulated pathways.


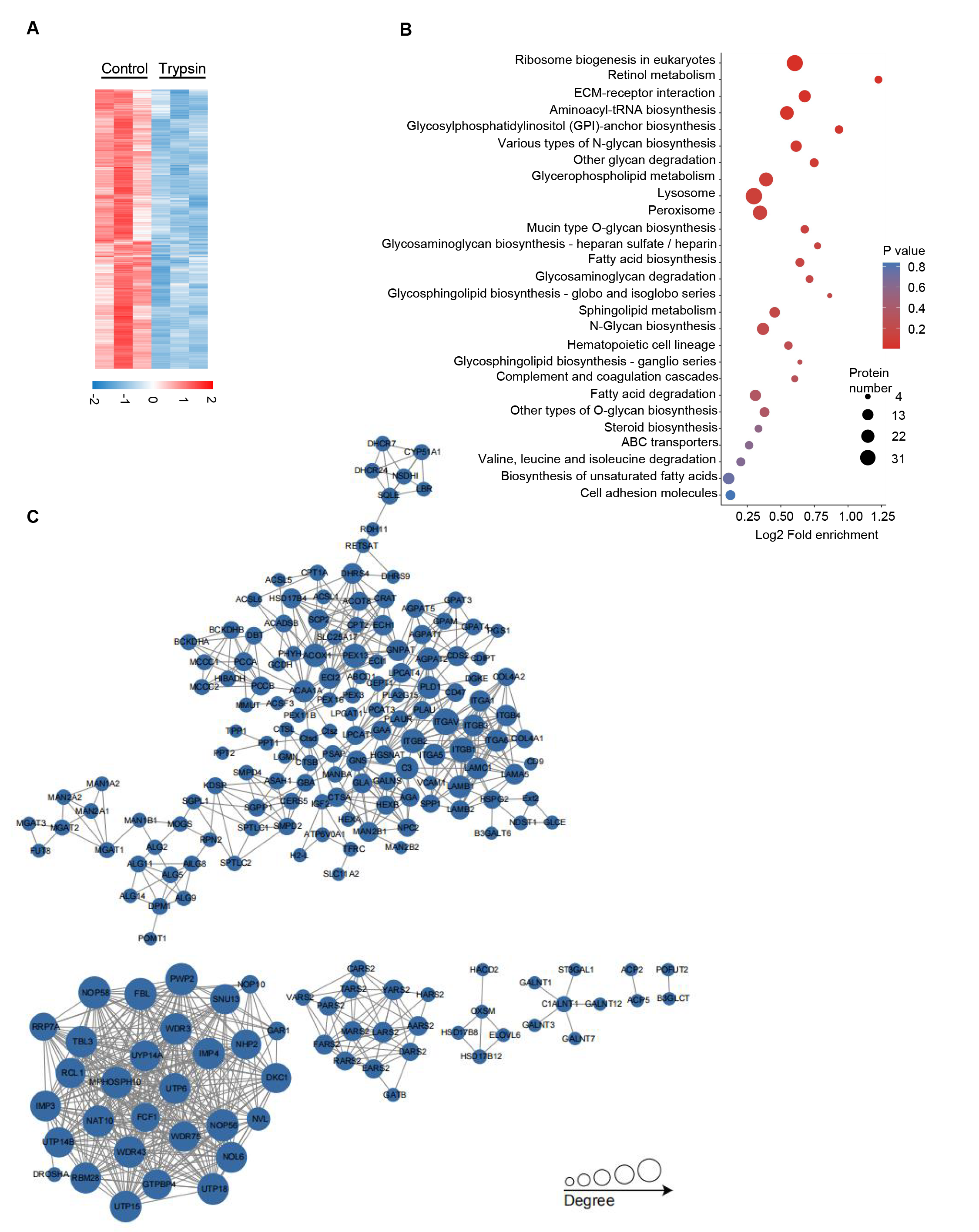


**Fig. S15** Proteomics analysis showing the down-regulated proteins in 4T1 cells after 0.5% trypsin treated for 30 min. (A) Heat map of down-regulated proteins. (B) KEGG enrichment pathways associated with tumor. (C) Cancer-associated proteins interaction network analysis.


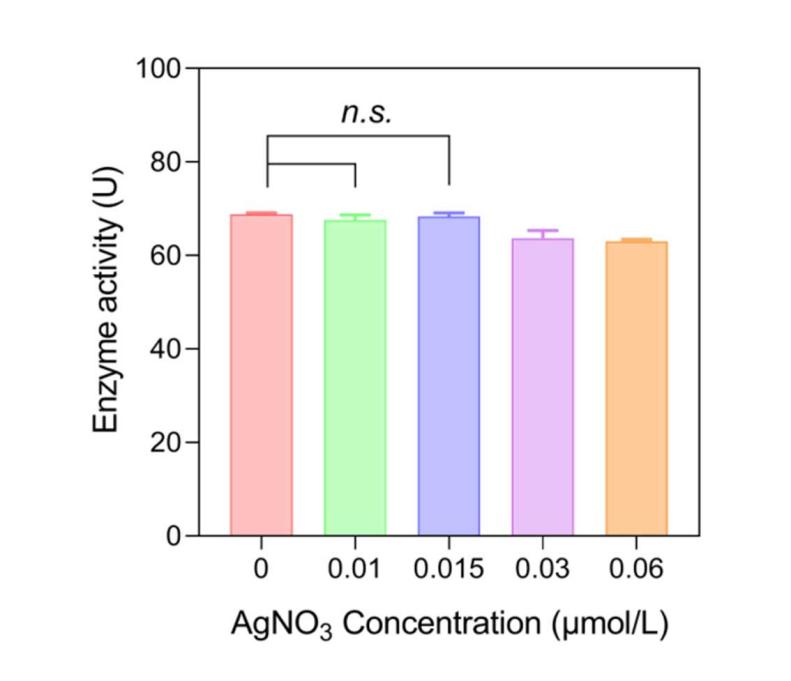


**Fig. S16** Influence of different concentrations of AgNO_3_ on trypsin activity. Low concentrations (0–0.015 µmol/L) of AgNO_3_ had little effect on trypsin activity. The experiments were conducted three times independently. *n.s.*, no significance.


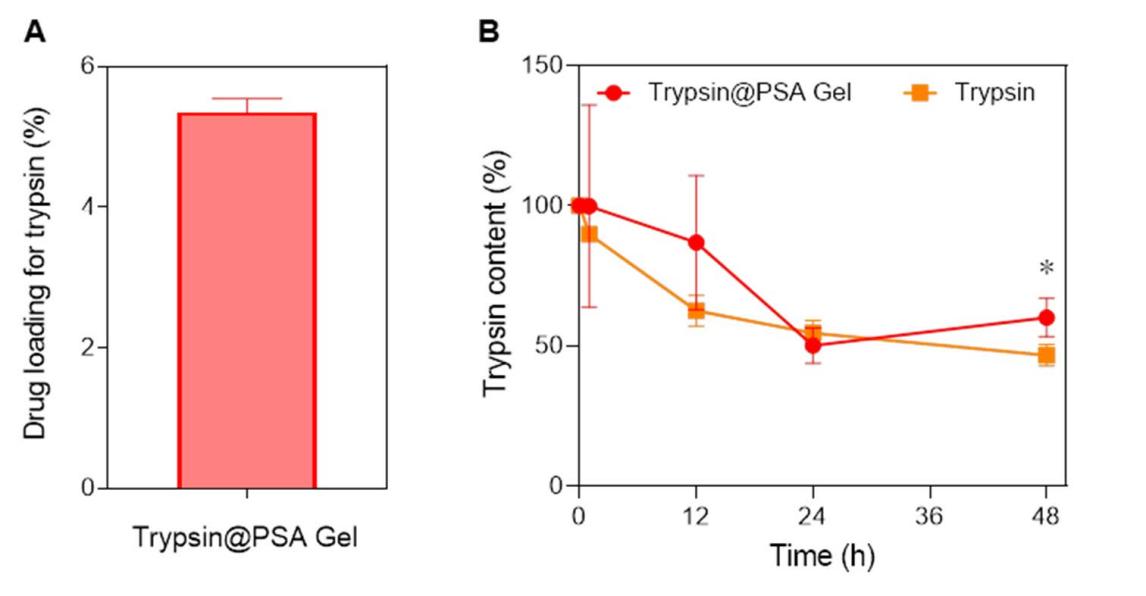


**Fig. S17** (A) The drug loading content of trypsin in Trypsin@PSA Gel. (B) Stability of trypsin in free form and Trypsin@PSA Gel at 4 ℃. The content and activity of trypsin remained unchanged after the formation of Trypsin@PSA Gel until 48 h. When compared with free trypsin, these findings (**Fig.** 1J and **Fig.** S3) indicate that Trypsin@PSA Gel prevented trypsin from deactivation after 48 h at 4 ℃ and 12 h at 37 ℃. The experiments were conducted three times independently. **p*<0.05.


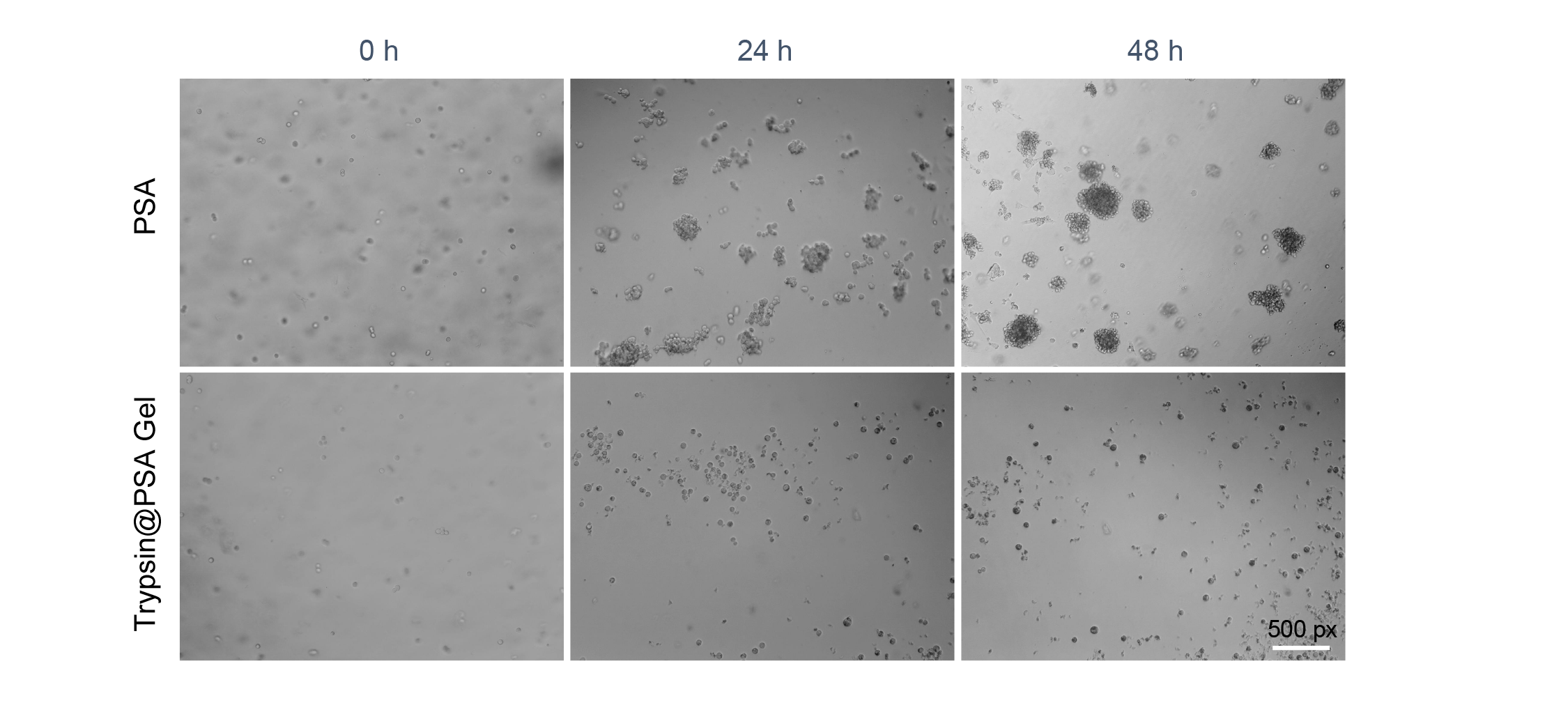
**Fig. S18** Representative images showing influence of PSA or Trypsin@PSA Gel on cell growth from 0 h to 48 h. Amplification: 100×. PSA enabled aggregative growth of 4T1 cells, whereas trypsin released from Trypsin@PSA Gel caused cell death by digesting the cells. These results demonstrate that PSA was not toxic for 4T1 cells. However, trypsin-containing PSA was toxic to 4T1 cells.


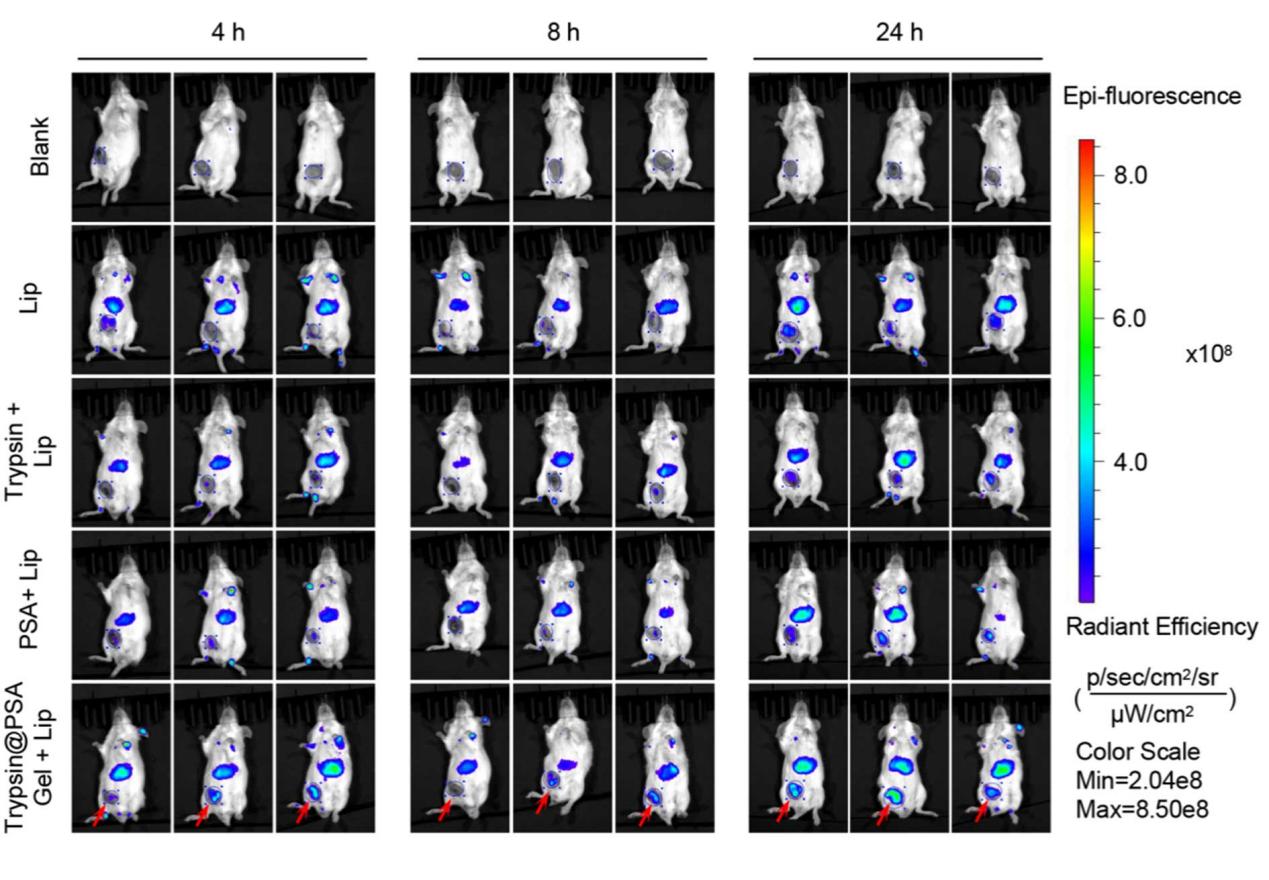


**Fig. S19** Comparison of DiD-Lip biodistribution in subcutaneously 4T1 tumor-bearing mice with and without trypsin digestive treatment. Red arrows indicate implanted tumors. The experiments were conducted three times independently.


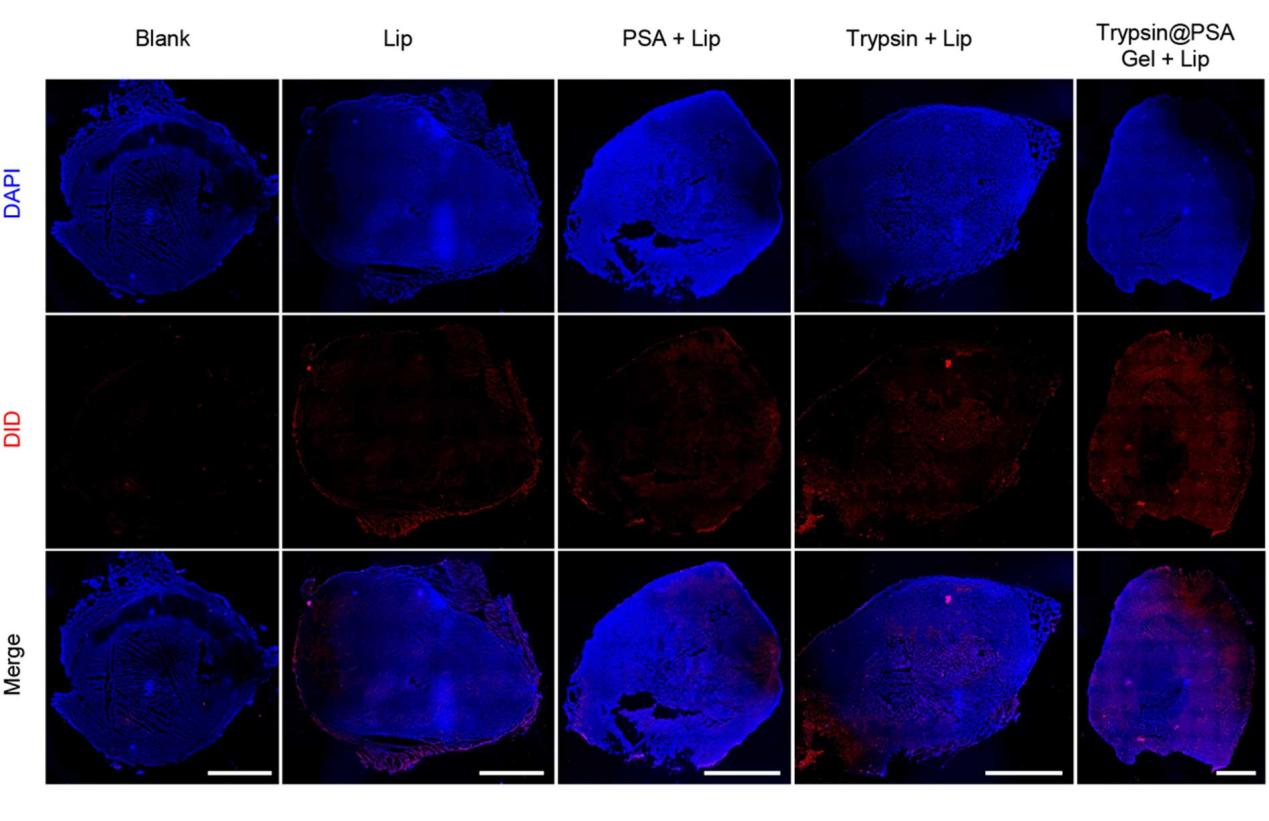


**Fig. S20** Penetration of DiD-Lip into tumors with or without trypsin digestive treatment. Whole tumors were collected for frozen sectioning and CLSM scanning. Scale bars = 2000 µm. DiD-Lip accumulated around the tumors in both the Lip and PSA groups without trypsin. However, when treated with trypsin in the form of free trypsin or Trypsin@PSA Gel, more DiD-Lip penetrated deeply into the centre of tumors. Furthermore, Trypsin@PSA Gel induced a more significant increase in red DiD-Lip signals within tumors than free trypsin.


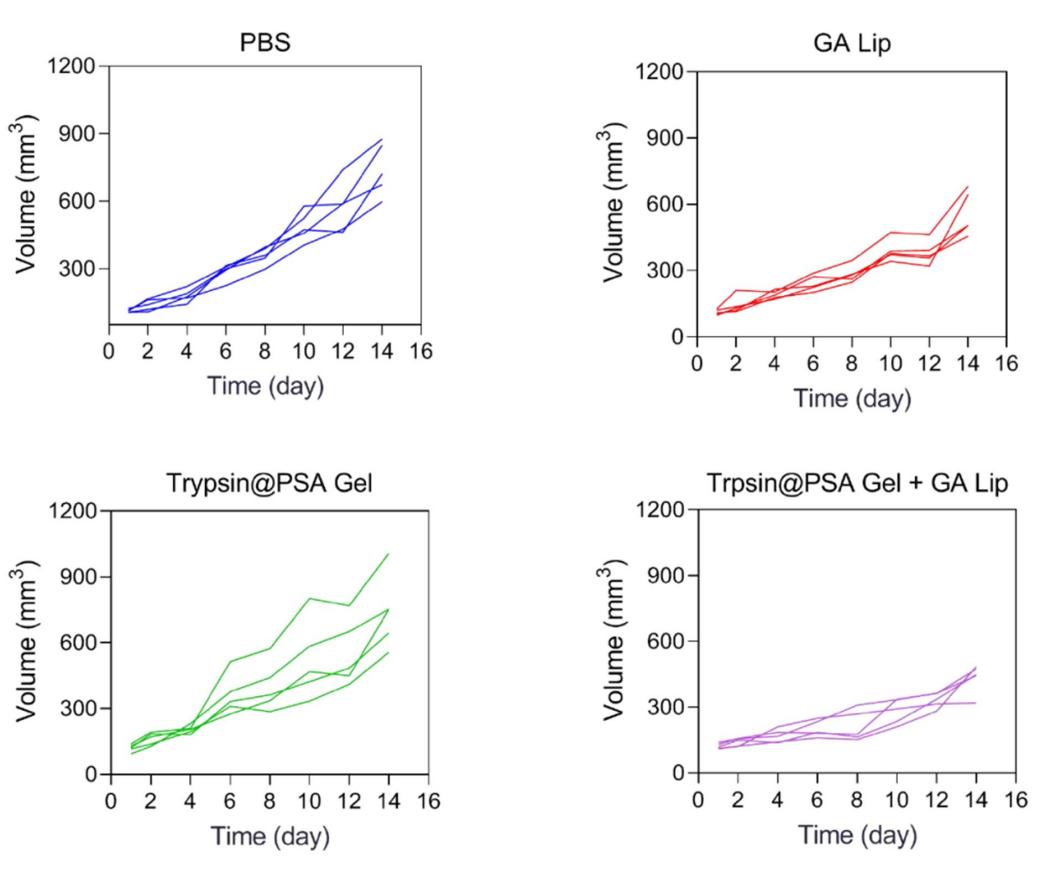


**Fig. S21** Individual tumor growth kinetics in response to the combination of GA-Lip chemotherapy and trypsin digestive therapy.


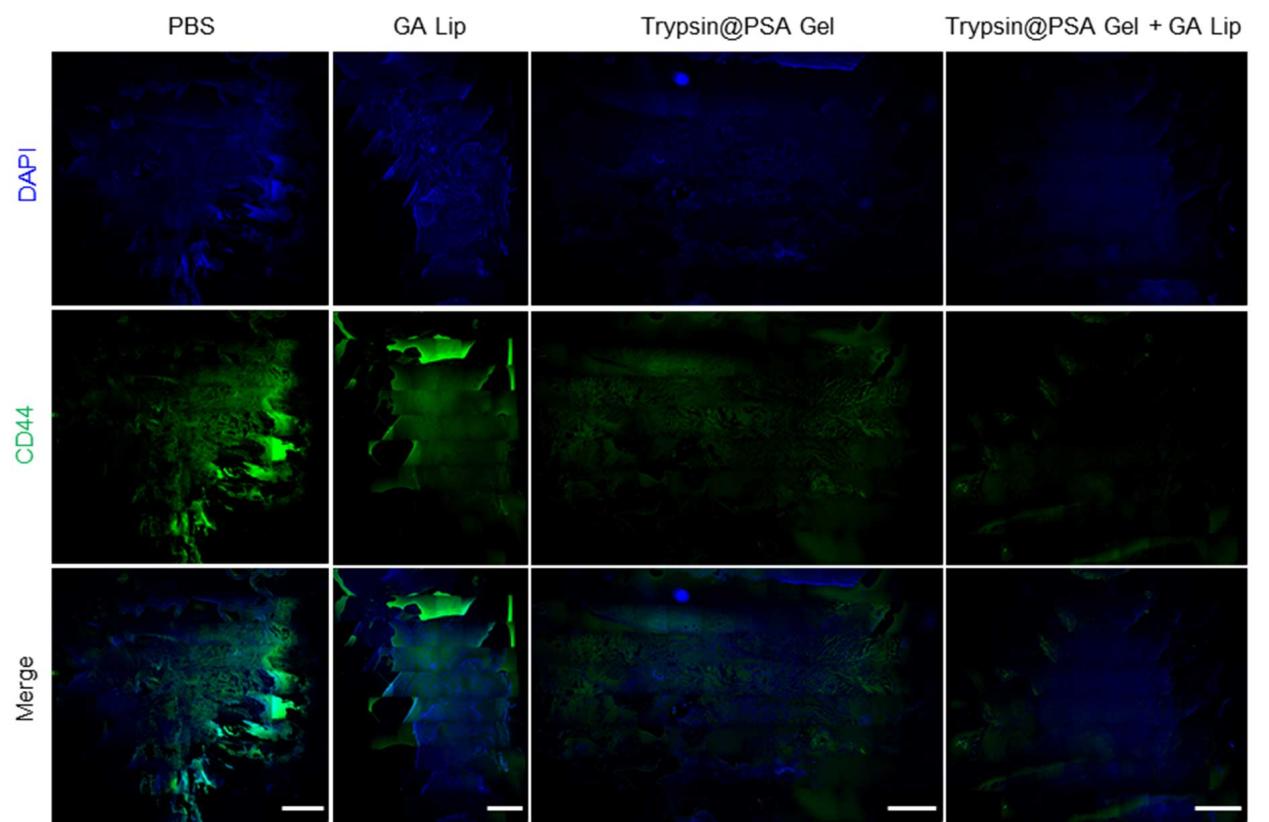


**Fig. S22** Immunofluorescence sections of the entire tumor with CD44 staining. Scale bars = 2000 µm. This result indicates that the combination of GA-Lip chemotherapy and trypsin digestive therapy inhibited the CD44 expression.


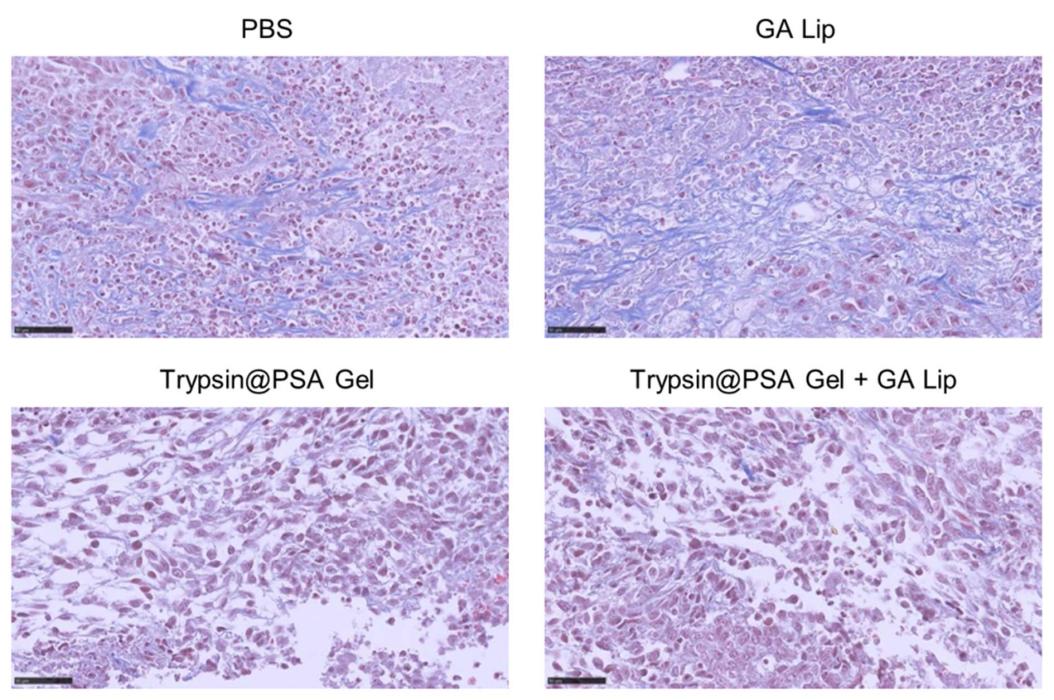


**Fig. S23** Masson’s trichome staining of tumors showing the collagen fibers after different treatments. Scale bars = 50 μm.


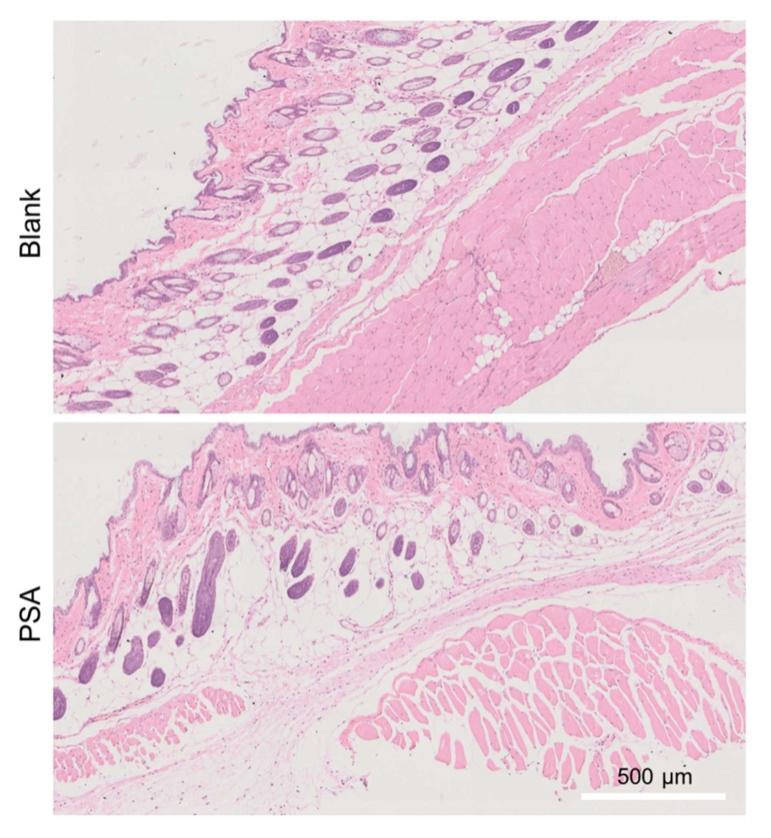


**Fig. S24** H&E-stained skin and muscle around the injection site. Scale bar = 500 μm.

**Supplementary Video 1** Tumorsphere treated by trypsin. Lava inspired the concept of trypsin-based digestive therapy for cancer.

**Supplementary Video 2** Tumor tissue treated by trypsin. Lava inspired the concept of trypsin-based digestive therapy for cancer.

**Supplemental references:**

[1] Q. Huang, S. Liu, Y. Tang. J Mol Biol 1993, 229: 1022-1036.
